# Skull remains of the dinosaur *Saturnalia tupiniquim* (Late Triassic, Brazil): With comments on the early evolution of sauropodomorph feeding behaviour

**DOI:** 10.1371/journal.pone.0221387

**Published:** 2019-09-06

**Authors:** Mario Bronzati, Rodrigo T. Müller, Max C. Langer

**Affiliations:** 1 Laboratório de Paleontologia, Faculdade de Filosofia Ciências e Letras de Ribeirão Preto, Universidade de São Paulo, Ribeirão Preto, São Paulo, Brazil; 2 Centro de Apoio à Pesquisa Paleontológica, Universidade Federal de Santa Maria, Santa Maria, Rio Grande do Sul, Brazil; University of Vienna, AUSTRIA

## Abstract

*Saturnalia tupiniquim* is a sauropodomorph dinosaur from the Late Triassic (Carnian–c. 233 Ma) Santa Maria Formation of Brazil. Due to its phylogenetic position and age, it is important for studies focusing on the early evolution of both dinosaurs and sauropodomorphs. The osteology of *Saturnalia* has been described in a series of papers, but its cranial anatomy remains mostly unknown. Here, we describe the skull bones of one of its paratypes (only in the type-series to possess such remains) based on CT Scan data. The newly described elements allowed estimating the cranial length of *Saturnalia* and provide additional support for the presence of a reduced skull (i.e. two thirds of the femoral length) in this taxon, as typical of later sauropodomorphs. Skull reduction in *Saturnalia* could be related to an increased efficiency for predatory feeding behaviour, allowing fast movements of the head in order to secure small and elusive prey, a hypothesis also supported by data from its tooth and brain morphology. A principal co-ordinates analysis of the sauropodomorph jaw feeding apparatus shows marked shifts in morphospace occupation in different stages of the first 30 million years of their evolutionary history. One of these shifts is observed between non-plateosaurian and plateosaurian sauropodomorphs, suggesting that, despite also having an omnivorous diet, the feeding behaviour of some early Carnian sauropodomorphs, such as *Saturnalia*, was markedly different from that of later Triassic taxa. A second shift, between Late Triassic and Early Jurassic taxa, is congruent with a floral turnover hypothesis across the Triassic-Jurassic boundary.

## Introduction

The first steps of sauropodomorph evolution are mainly known based on the fossil record of two South American Carnian deposits, the Santa Maria (c. 233 Ma) and the Ischigualasto (c. 231 Ma) formations of Brazil and Argentina, respectively [1,2]. In early 1998, three skeletons were unearthed during two fieldwork campaigns in the locality commonly known as Cerro da Alemoa or Waldsanga (53°45’ W; 29°40’ S), located in the outskirts of Santa Maria, south Brazil, in the red mudstones of the Santa Maria Formation. These skeletons were assigned to a new species of sauropodomorph dinosaur, *Saturnalia tupiniquim* [3], which was at the time the oldest known member of the group. For more than twenty years, the only cranial elements available for *Saturnalia* (from one of its paratypes) were the frontals, the left squamosal and postorbital, and the braincase, which were preserved exposed on the rock surface of the same block, along with isolated right lacrimal and left dentary. In the absence of detailed descriptions, some phylogenetic analyses (e.g. [4,5,6]) incorporated information collected first-hand from those partially exposed elements in their data matrices. In 2014, a Computed Tomography procedure revealed that the parietals and laterosphenoids were preserved inside the matrix of the same block containing the braincase elements. Later, in 2016, during further preparation of MCP-3845-PV, additional cranial bones were discovered underneath the pelvic girdle of the specimen, including left quadrate, prefrontal, and lacrimal, as well as partial left maxilla and right dentary. Herein, we describe all the available skull bones of *Saturnalia*, except for the braincase, described elsewhere (see [7]). Based on this new information, alongside recent fossil findings [8–11] and a new principal co-ordinates analysis, we provide new insights on the early evolution of the sauropodomorph feeding behaviour.

## Systematic Terminology

Here we follow the definitions of [12] for Sauropodomorpha (the most inclusive clade containing *Saltasaurus loricatus*, but not *Passer domesticus* or *Triceratops horridus*), Plateosauria (the most recent common ancestor of *Plateosaurus engelhardti* and *Jingshanosaurus xinwaensis*, and all its descendants), and Anchisauria (the most recent common ancestor of *Anchisaurus polyzelus* and *Melanorosaurus readi*, and all its descendants), of [13] for Massopoda (the most inclusive clade containing *Saltasaurus loricatus* but not *Plateosaurus engelhardti*), and of [14] for Sauropoda (the least inclusive clade containing *Vulcanodon karibaensis* and Eusauropoda).

## Material and methods

### Referred material and justification

MCP-3845-PV: partial left maxilla, both frontals, parietals, lacrimals, postorbitals, and dentaries, left quadrate and prefrontal, apart from the braincase [7] and a fairly complete postcranial skeleton [15] to be described elsewhere. All the cranial elements were preserved disarticulated, except for those of the braincase [7]. Nevertheless, they can be safely assigned to MCP-3845-PV, because they were found in close association with the postcranial material of this specimen, which is isolated from other dinosaur skeleton found in the area (but see Discussion below). Furthermore, the bones match in relative sizes and there are no duplicated elements.

### CT-Scan

A virtual preparation using computed tomography was preferred because the bones are preserved in heavily fractured blocks, in such a way that mechanical preparation could damage the fossils. The block containing the frontals and parietals was scanned at the Zoologische Staatsammlung München (Munich, Germany) and those including other bones at the Centro para Documentação da Biodiversidade, Universidade de São Paulo (Ribeirão Preto, Brazil). In both occasions, the scan was conducted in a Nanotom Scan machine—GE Sensing & Inspection Technologies GmbH, Wunstorf Germany. The slices generated were manually segmented in the software Amira (version 5.3.3, Visage Imaging, Berlin, Germany).

### Description

Main taxa used for comparisons and the corresponding information source (first-hand analysis and/or literature) are as follows: *Adeopapposaurus mognai* (PVSJ 568; PVSJ 610); *Buriolestes schultzi* (CAPPA/UFSM 0035); *Eoraptor lunensis* (PVSJ 512); *Massospondylus* spp. (SAM-PK-K1314; [16]); *Panphagia protos* (PVSJ 8743); *Plateosaurus* spp. (SMNS *13200*; [17]). For the following comparisons, sources of anatomical data (specimens indicated by collection number and/or previous studies) are provided only if different from those listed above.

### Skull length estimate

The cranial length of *Saturnalia* was here estimated based on the length of the right frontal of MCP-3845-PV, which is more complete than the left element. Skull size was inferred with the aid of linear regressions, based on measurements of other taxa known from more complete specimens (see details in the Results section below). Our estimates employed a value 15% greater than the preserved length (length as preserved = 29 mm) of the frontal, in order to account for uncertainties regarding the completeness of the anterior margin of the bone (see description below). Linear regressions were also employed to estimate the mandible length of *Saturnalia*, based on the preserved dentaries of MCP-3845-PV. In this case, the distance from the anterior tip of the mandible to the anterior margin of the external mandibular fenestra was used as a proxy to estimate the length of the lower jaw, also based on values measured for other taxa (see Results and Discussion Below). Neither of the dentaries of *Saturnalia* is completely preserved. The left element is 51 mm long from the anterior end of the mandibular fenestra to the mesial margin of the anterior most preserved tooth, which is likely not at the very anterior tip of the dentary (see description below). The right element is c. 44 mm long as preserved, and it was probably not much longer based on the dentition pattern along its anteroposterior axis. Accordingly, to account for the uncertainties regarding the length of the dentary, we estimated a range of values between 44–59,3 mm for the length between the anterior tip of the bone to the anterior margin of the mandibular fenestra (see Discussion below).

### Phylogenetic analyses

Here, for the first time, all cranial anatomy data available for *Saturnalia* was used to assess the phylogenetic relationships of the taxon. We conducted two phylogenetic analyses, using expanded and modified versions of the data matrices of [8], focused on early dinosauromorphs, and [18], focused on non-neosauropodan sauropodomorphs (S1 Appendix). The resulting data matrices were analysed using TNT v. 1.1 [19] via heuristic searches under the following parameters: 1000 replicates of Wagner Trees, hold 10, TBR (tree bi-section and reconnection) for branch swapping. A second round of TBR was conducted using the Most Parsimonious Trees (MPTs) recovered in the first interaction of each analysis.

### Principal co-ordinates analysis

We investigated the morphospace (= discrete character space) occupation of cranial features associated with the feeding apparatus of sauropodomorphs using Principal co-ordinates analysis (PCoA) based on Maximum Observed Rescaled Distances [20,21] implemented in the R package Claddis [21]. The discrete character taxon-matrix used in the analyses consisted of a reduced matrix derived from that modified from [18], using only characters related to the jaw feeding apparatus (S1 Appendix), including dentition (37 out of the 412 characters used for the phylogenetic analysis). Only taxa possessing less than 50% of missing data for the corresponding characters were included in the analyses in order to reduce non-comparability problems. Additionally, we used nonparametric multivariate analysis of variance (npMANOVA [22]) implemented in the R package RVAideMemoire [23] to test for significant differences in the distribution of groups in the morphospace. A total of 10.000 permutations using all PC scores and BH correction [24] were conducted.

## Results

### Description

#### Maxilla

**Fig 1:**
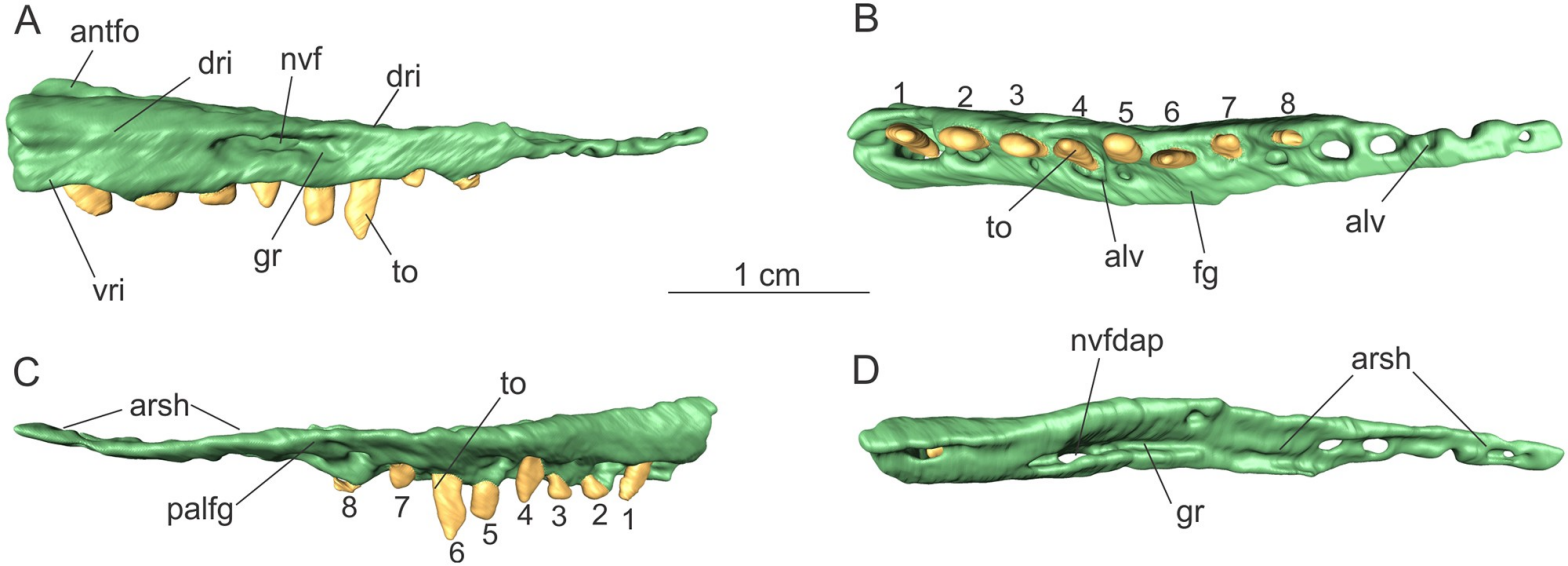
Posterior ramus of the right maxilla of the specimen MCP-3845-PV of *Saturnalia tupiniquim* in lateral (A), ventral (B), medial (C), and dorsal (D) views. Abbreviations: **alv—**alveolous; **antfo—**antorbital fossa; **arsh—**articulation shelf; **dri—**dorsal ridge on the lateral surface; **fg—**flange; **gr—**groove; **nvf—**neurovascular foramina; **to—**tooth; **nvfdap—**dorsal aperture assocaied to the neurovascular foramen; **palfg—**palatine flange; **vri—**ventral ridge on the lateral surface.

Only part of the posterior ramus of the left maxilla was found, with an anteroposterior length of 34 mm as preserved (Fig 1). The posterior ramus comprises an anteroposteriorly elongated rod-like structure, gently tapering posteriorly in lateral view. The posterior third is dorsoventrally flat, forming a shelf (“arsh” in Fig 1) that would receive the rostral process of the jugal dorsally.

A gentle lateral expansion of the alveolar margin is seen on the lateral surface of the posterior process (‘vri’ in Fig 1). This expansion extends along the entire series of preserved teeth, anterior to the dorsoventrally flattened portion of the process. Similarly, the dorsal margin of the ramus also expands laterally, as a faint ridge (‘dri’ in Fig 1) extends anteroposteriorly in this area over the lateral surface. The surface between the dorsal and ventral ridges is dorsoventrally concave. A large foramen (‘nvf’ in Fig 1) pierces the lateral surface of the bone, but its size (= 3.6 mm long anteroposteriorly) might have been exaggerated due to poor preservation. We identify this foramen as the posterior most neurovascular foramen of the lateral surface of the maxilla, which in sauropodomorphs is typically larger than more anterior maxillary foramina [4,25,26]. An elliptical groove is associated with this foramen, also resembling the condition seen in other sauropodomorphs. In the dorsal surface of the posterior process, an opening (‘nvfdap’ in Fig 1) is associated with that neurovascular foramen. Posterior to the opening, there is an anteroposteriorly elongated groove, which extends until the articulation with the jugal, as also observed in *Pl*. *erlenbergensis* (AMNH 6810).

The ventral and dorsal margins of the medial surface of the maxilla above the tooth line are respectively horizontal and anterodorsally to posteroventrally inclined (Fig 1) in the anterior portion of the posterior process (as preserved). Anteriorly, the medial surface is c. 4 mm dorsoventrally deep, but it tapers distally, with its dorsal margin merging with the ventral anterior to the articular shelf for the jugal. In this area, the medial surface of the maxilla exhibits a flange (‘fg’ in Fig 1), which is interpreted as the surface contacting the palatine medially.

There are 13 tooth positions, suggesting a high count of maxillary teeth as in other sauropodomorphs such as *Buriolestes*, *Pampadromaeus barbarenai*, *Eoraptor*, and *Plateosaurus* spp. On the anterior part of the preserved portion of the bone, a dorsal expansion of the medial margin forms a transversely thin ridge. The lateral surface of this ridge bears a depression, which is interpreted as the posterior end of the maxillary antorbital fossa (‘antfo’ in Fig 1). This fossa extends for no more than one fourth of the total anteroposterior length of the preserved part of the posterior ramus of the maxilla.

#### Frontal

**Fig 2:**
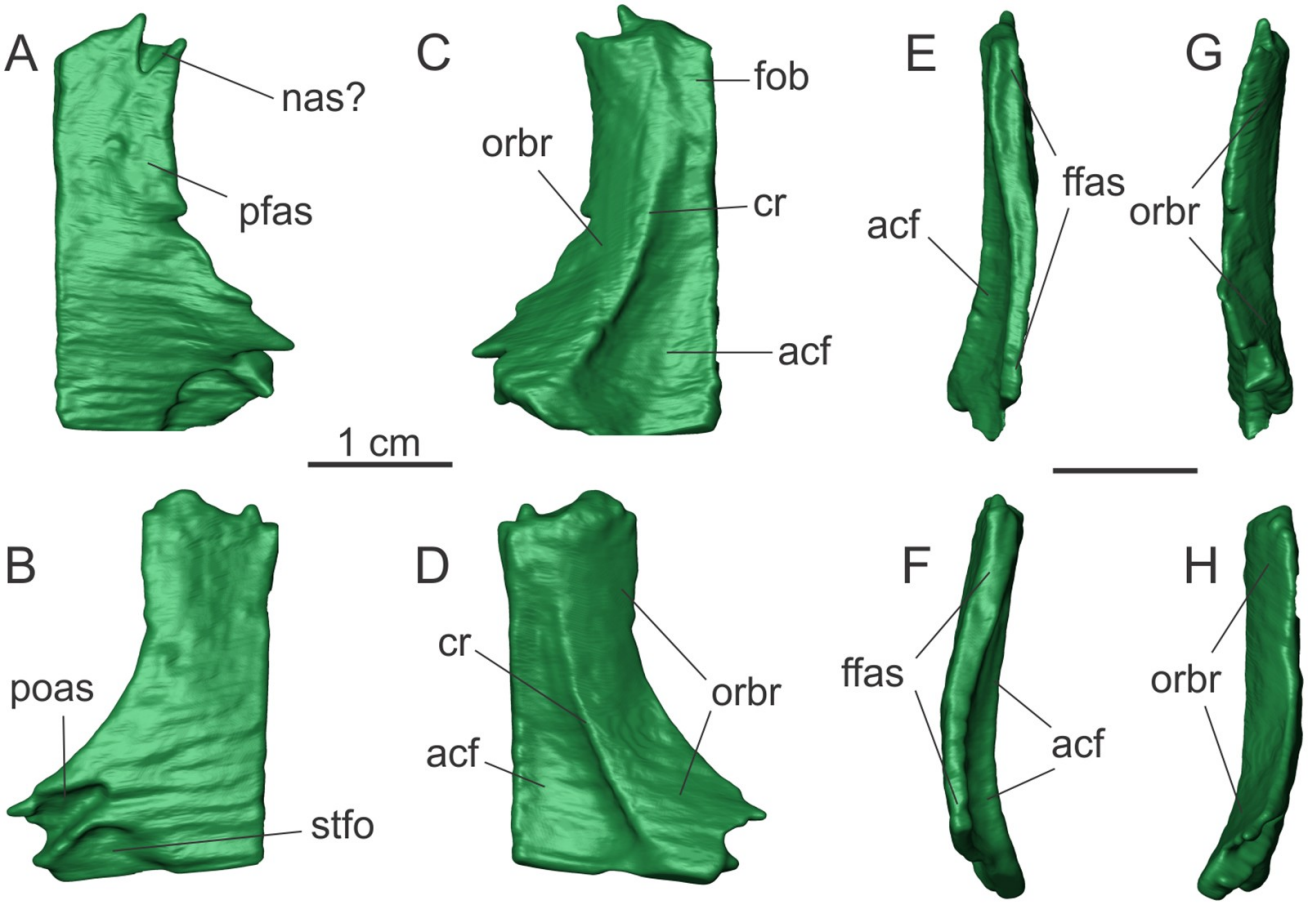
Right and left frontals of the specimen MCP 3845 PV of *Saturnalia tupiniquim* in dorsal (A, B), ventral (C, D), medial (E, F), and lateral (G, H) views, respectively. Abbreviations: **acf—**anterior cranial fossa; **cr—**crest; **ffas—**frontal/frontal articulation surface; **fob—**fossa for the olfactory bulb; **nas**—articulation surface with the nasal; **orbr—**orbital roof; **pfas—**articulation surface with the prefrontal; **poas—**articulation surface with the postorbital; **stfo—**supratemporal fossa.

CT-scan data shows both frontals of MCP-3845-PV are preserved inside the matrix (Fig 2). Each bone is arched dorsally in the anteroposterior axis, with the most dorsal point approximately at the mid-length of the bone. This results in a concave ventral surface in lateral/medial views. Even probably lacking a small part of its anterior tip (see Discussion below), the frontal is c. 1.7 times longer than wide (maximal length estimated in ca. 29–30 mm; maximal width ca. 17 mm). Martinez et al. [27] stated that some sauropodomorphs, such as *Pl*. sp., *Adeopapposaurus*, and *Massospondylus* spp. possess a frontal that is wider than long, differing from the condition of most Carnian dinosaurs. However, the frontals of *Plateosaurus*. spp., *Adeopapposaurus*, and *Massospondylus* spp. are also longer than wide. The width is only greater than the length if the measurement is taken from both frontals together. The articulated frontals of MCP-3845-PV form a sub-rectangular anterior half and a T-shaped outline in dorsal/ventral views (Fig 2). From its mid-length, each frontal becomes progressively wider posteriorly.

A slot in the anterolateral corner of the dorsal surface of the right frontal might represent the facet for the articulation with the nasal (‘nas’ in Fig 2), but could also be an artefact related to a breakage of the anterior margin, given that the structure is not so clear in the left bone. Slightly posterior to this notch, a shallow, half-moon shaped depression is seen on the right frontal (‘pfas’ in Fig 2), which likely represents the surface of the frontal that was overlapped by the prefrontal. This depression extends for slightly more than one third of the anteroposterior length of the frontal and medially reaches half of the width of the bone in this region. Still on the dorsal surface of the frontal, the articulation area for the postorbital is located in its posterolateral corner (‘poas’ in Fig 2), anterolateral to the portion of the frontal that contributes to the anterior margin of the supratemporal fossa. The slot for the postorbital extends anteromedially and is separated by a crest from the supratemporal fossa. That fossa (‘stfo’ in Fig 2) occupies the lateral-half of the posterior margin of the frontal, and assumes the shape of a half-moon in dorsal view.

The posterior margin of the frontal is lateromedially straight to slightly convex in dorsal view (Fig 2). The frontal does not participate in the border of the supratemporal fenestra, but is excluded from that by an anterolateral projection of the parietal (see below) that likely contacted the laterosphenoid on the anterior margin of the fenestra. This condition is also observed in other sauropodomorphs such as *Buriolestes*, *Plateosaurus* spp, and *Massospondylus* spp. The participation of the frontal in the supratemporal fenestra of early sauropodomorphs has been recently discussed [27]. Based on our observations of *Panphagia*, we consider that the irregular shape of the posterior margin of its frontals is most likely due to breakage, and it is not possible to be sure about the presence of a triangular posterior projection reaching the fenestra (see Figure 2 in [27]).

In *Saturnalia*, a crest extends along the entire anteroposterior axis of the ventral surface of the frontal (‘cr’ in Fig 2), setting two distinct surfaces apart, the orbital roof laterally (Fig 2) and the endocranial surface medially. This configuration is also seen in the sauropodomorphs *Efraasia minor* and *Plateosaurus* spp. A different condition is observed in *Panphagia*, in which two parallel ridges separate the two regions [27].

The lateral margin of the frontal is formed by the surface corresponding to the roof of the orbit (Fig 2). This is more dorsally raised at its midpoint, following the general condition of the entire bone. Thus, the orbital roof is dorsally arched in lateral view. Likewise, it is ventrally concave in transverse section, raising dorsally towards the lateral margin at an angle of ca. 45 degrees to the endocranial roof. In ventral view, the lateral margin of the orbital roof parallels the crest that forms its medial limit (as defined above). Hence, the lateromedial width of the orbital roof remains constant along its entire anteroposterior length. Also, the orbital roof extends along the entire lateral margin of the frontal as preserved.

Two distinct fossae are present on the endocranial surface of the more complete right frontal (Fig 2). The more posterior probably represents the anterior cranial fossa (*fossa cranii anterioris* in [27]–‘acf’ in Fig 2), where the frontal roofed part of the anterior portion of the brain (telencephalon). That fossa extends for ca. 75% of the anteroposterior length of the frontal, reaching the posterior margin of the bone. It occupies most of the endocranial surface of the frontal, except for its lateroposterior corner, where the ventral surface of the bone is flat. Anterior to the *fossa cranii anterioris*, in the anterior fourth of the frontal, another depression corresponds to the fossa for the olfactory bulb (‘fob’ in Fig 2). This is elliptical in shape and approximately five times smaller than the anterior cranial fossa.

#### Parietal

**Fig 3:**
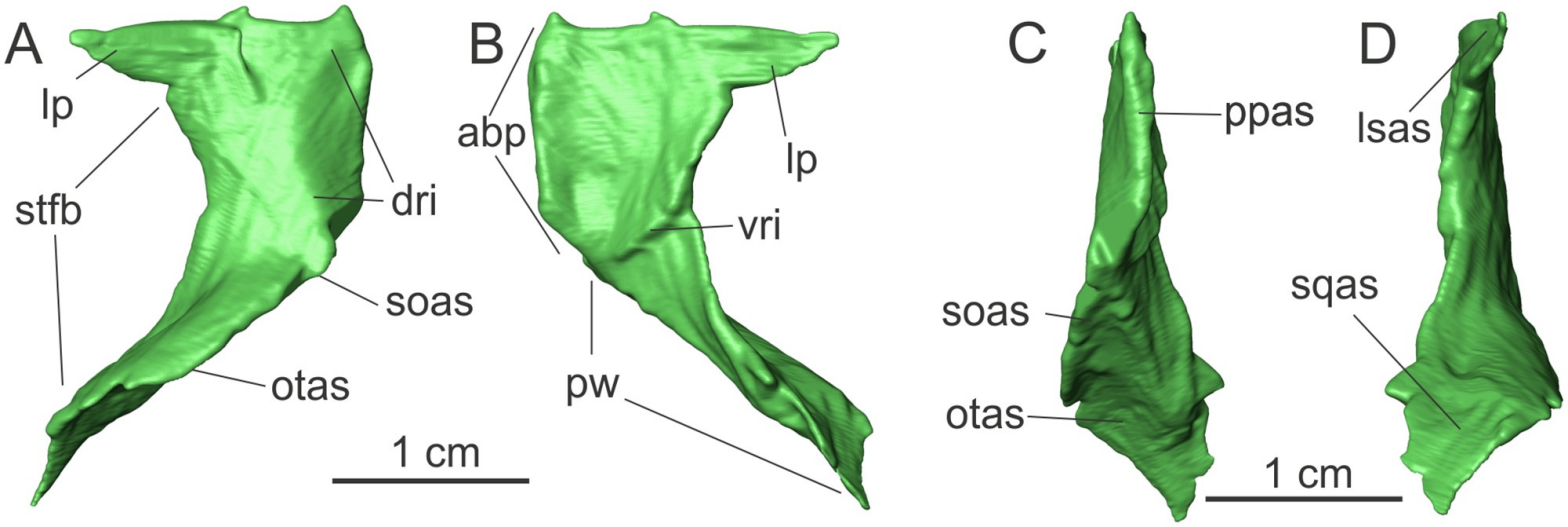
Left parietal of the specimen MCP 3845 PV of *Saturnalia tupiniquim* in dorsal (A), ventral (B), medial (C), and lateral (D) views. Abbreviations: **abp**—anterior body of the parietal; **dri—**dorsal ridge; **lp—**lateral projection; **lsas—**articulation surface with the laterosphenoids; **otas—**articulation surface with the otoccipital; **ppas—**parietal/parietal articulation surface; **pw—**parietal wing; **soas—**articulation surface with the supraoccipital; **sqas—**articulation surface with the squamosal; **stfb—**border of the supratemporal fenestra; **vri—**ventral ridge.

The description of the parietal is based only on the left element, which is completely preserved (Fig 3). The right parietal is broken and partially preserved in separate pieces, adding no extra anatomical information. The total length of the bone is ca. 22 mm, and its maximum width, from the posterolateral corner of the wing to the medial articulation to the counterpart, is 13 mm. The parietal is composed of two parts, the anterior body (‘abp’ in Fig 3), sometimes treated as the “main body” (e.g. [27]), and the parietal wing (‘pw’ in Fig 3). The former corresponds to the portion extending from the anterior margin of the bone to the point where its transverse axis is twisted from a horizontal to a vertical plane. The parietal is isolated, but its anterior body probably contacted the frontal anteriorly, the supraoccipital posteromedially, the laterosphenoid lateroventrally, and possibly the postorbital laterally. The parietal wing would have contacted the supraoccipital medially, the paraoccipital process of the otoccipital ventrally, and the squamosal distally. In *Saturnalia*, the anteroposterior lengths of those two regions are nearly the same, as seen in *Eoraptor lunensis*. In contrast, the anteroposterior length of the anterior body is ca. 0.8 of that of the parietal wing in *Plateosaurus* spp, and ca. 1.5 in *Panphagia*.

The anterior margin of the parietal is mostly straight (the same is true for the posterior margin of the frontal), but bears two slots that may represent articulation points with posterior projections of the frontal margin (Fig 3). The absence of clearer indicatives of an interdigitating suture between parietals and frontals could be a preservation bias. The bones are not preserved in articulation and the small and delicate projections of an interdigitating suture could have been lost during preservation. Another possibility is that the CT-Scan data did not allow reconstructing the delicate morphology of an interdigitating suture, as suggested for *Lesothosaurus diagnosticus* [28].

The anterior body of the parietal has a triangular lateral projection at its anterolateral corner (‘lp’ in Fig 3). It is 5 mm long anteroposteriorly, which is about one fourth of the anteroposterior length of the anterior body of the parietal. The anterior margin of the projection is mostly straight, corresponding to one third of the total anterior width of the parietal. The projection is subtriangular in dorsal/ventral views and its anteroposterior axis is oblique to the horizontal, with the posterior margin being more ventrally positioned than the anterior. Its dorsal surface is part of the supratemporal fossa, and would be continuous with the part of the fossa entering the frontal, as described above. When the parietals and frontals are virtually articulated, the lateral projection of the former reaches the postorbital slot in the posterolateral margin of the latter (S2 Appendix). Thus, we infer that the frontal was excluded from the margin of the internal supratemporal fenestra.

The dorsal surface of the anterior body of the parietal, excluding the anterolateral projection, is transversally convex and roughly sub-rectangular in shape (Fig 3), with a concave lateral margin and a retracted posteromedial corner (but this might be an artefact due to breakage). A low laterally-arching ridge (‘dri’ in Fig 3) extends lateroposteriorly from the anteromedial corner of the bone until half the length of the anterior body of the parietal and then turns medioposteriorly towards its posteromedial corner. This results in a half-moon shape for the portion of the parietal medial to the ridge, which forms the skull roof. The anterior part of this ridge marks the medial limit of the supratemporal fossa on the parietal. The ridge projects more dorsally than the medial margin of the anterior body of the parietal. Thus, with the parietals articulated, the dorsal surface of the pair, between the ridges, is depressed.

In ventral view, the surface of the anterior body of the parietal is transversely and anteroposteriorly concave, mainly following the corresponding convexity of the dorsal surface of the bone (Fig 3). A posteromedially to anterolaterally oriented ridge (‘vri’ in Fig 3) separates the anterior body from the parietal wing. The ridge arches slightly posterolaterally and continues anteriorly to form part of the lateral margin of the parietal, reaching the posterior limit of the subtriangular anterolateral projection.

The parietal wing is a tall, posterolaterally extending lamina (Fig 3). Its anteroposterior length is ca. 10 mm, but the long axis is ca. 18 mm. The ventral and dorsal margins parallel one another for ca. 90% of the long axis of the process, but the latter descents ventrally at the distal tip, approaching the ventral margin, which remains at about the same dorsoventral level. The lateral surface of the parietal wing forms the medial and posteromedial margin of the supratemporal fossa. Its ventral half is dorsoventrally concave, following the shape of the ventral portion of the anterior body of the parietal that forms the supratemporal fossa. The lateral portion of this concave surface represents the articulation area with the parietal ramus of the squamosal, where both bones joined to form the posterior margin of the supratemporal fenestra. A low ridge marks the dorsal limit of that concave region, dorsal to which the lateral surface of the parietal wing is dorsoventrally convex. Based on the shape of the lateral surface of the parietal, it is very likely that the supratemporal fenestra was longer than wide in *Saturnalia* (S2 Appendix).

#### Prefrontal

**Fig 4:**
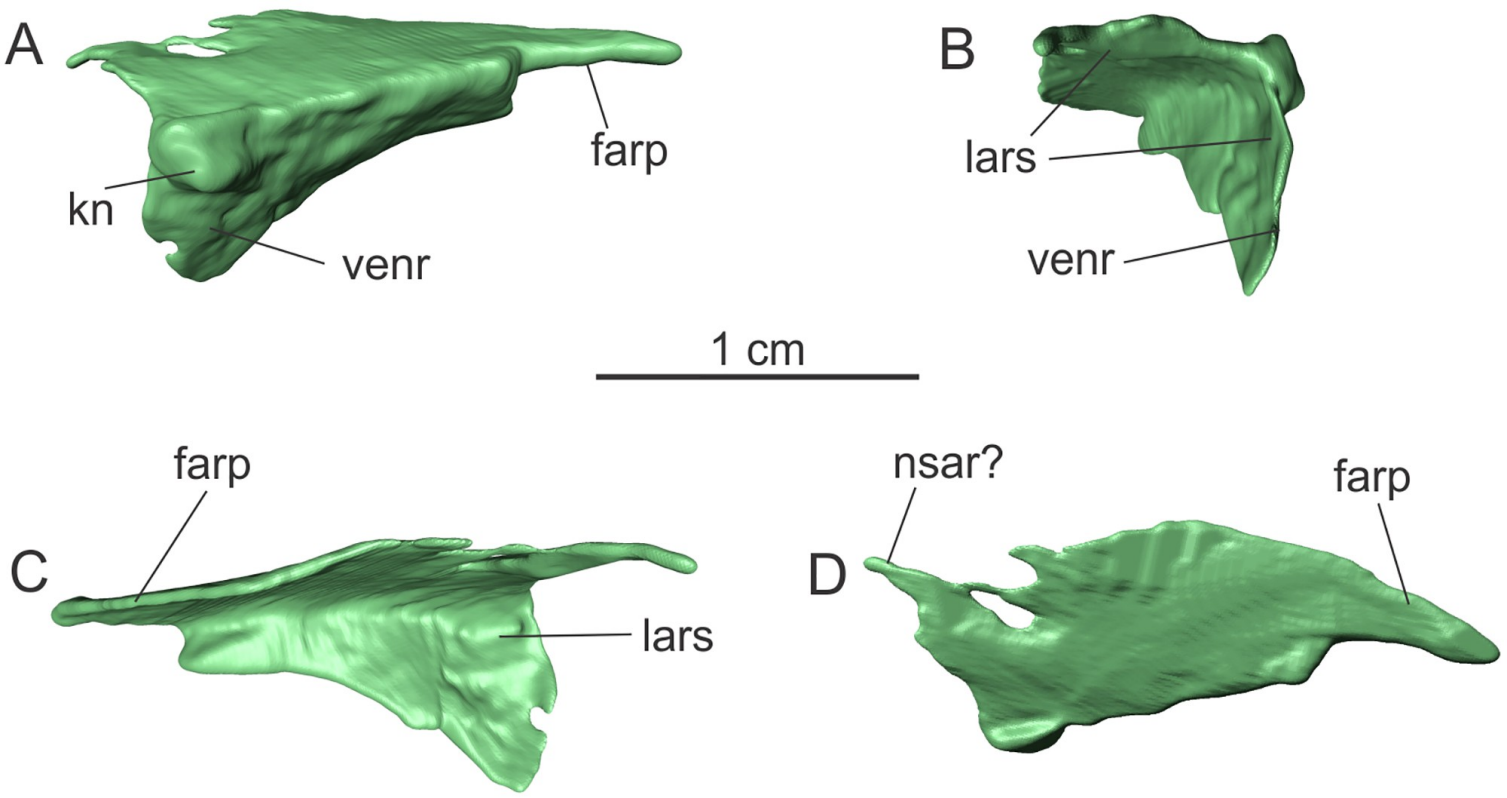
Left prefrontal of the specimen MCP-3845-PV of *Saturnalia tupiniquim* in lateral (A), anterior (B), medial (C), and dorsal (D) views. **Abbreviations**: **farp—**posterior process for the articulation with the frontal; **kn—**knob; **lars—**articulation surface with the lacrimal; **nsar—**articulation surface with the nasal; **venr—**ventral ramus.

Only the left prefrontal is preserved (Fig 4). The bone is almost complete, but lacks the distalmost part of the ventral ramus (‘venr’ in Fig 4), and the anterolateral margins is slightly fractured. The bone can be divided in a dorsoventrally flattened dorsal portion, and a mediolaterally thin ventral portion, which forms part of the orbital rim. In lateral view, a sharp edge marks the boundary between the dorsal and lateral portions. The medial surface of the bone is concave, mirroring the shape of the lateral side. The anterior margin of the dorsal and ventral portion meets at a right angle, so that the prefrontal is ‘L-shaped’ in anterior view (Fig 4B).

The lateral margin of the dorsal surface is mostly straight. Its posterior portion terminates in a finger-like process (‘farp’ in Fig 4), which is the portion of the bone that contacted the anterolateral corner of the frontal. Anteriorly, the dorsal surface of the prefrontal also terminates in a finger-like anteromedial projection, which might have contacted the nasal (‘nsar’ in Fig 4). The lateral surface of the ventral portion of the prefrontal is slightly concave anteroposteriorly, corresponding to anterodorsal margin of the orbit. A knob-like structure (‘kn’ in Fig 4) is present on the anterodorsal corner of the ventral ramus of the prefrontal, as also observed in *Panphagia*. A groove on the medial surface of the bone, adjacent to the knob on the lateral side, likely represents the area of contact with the dorsal surface of the lacrimal (‘lars’ in Fig 4).

#### Lacrimal

**Fig 5:**
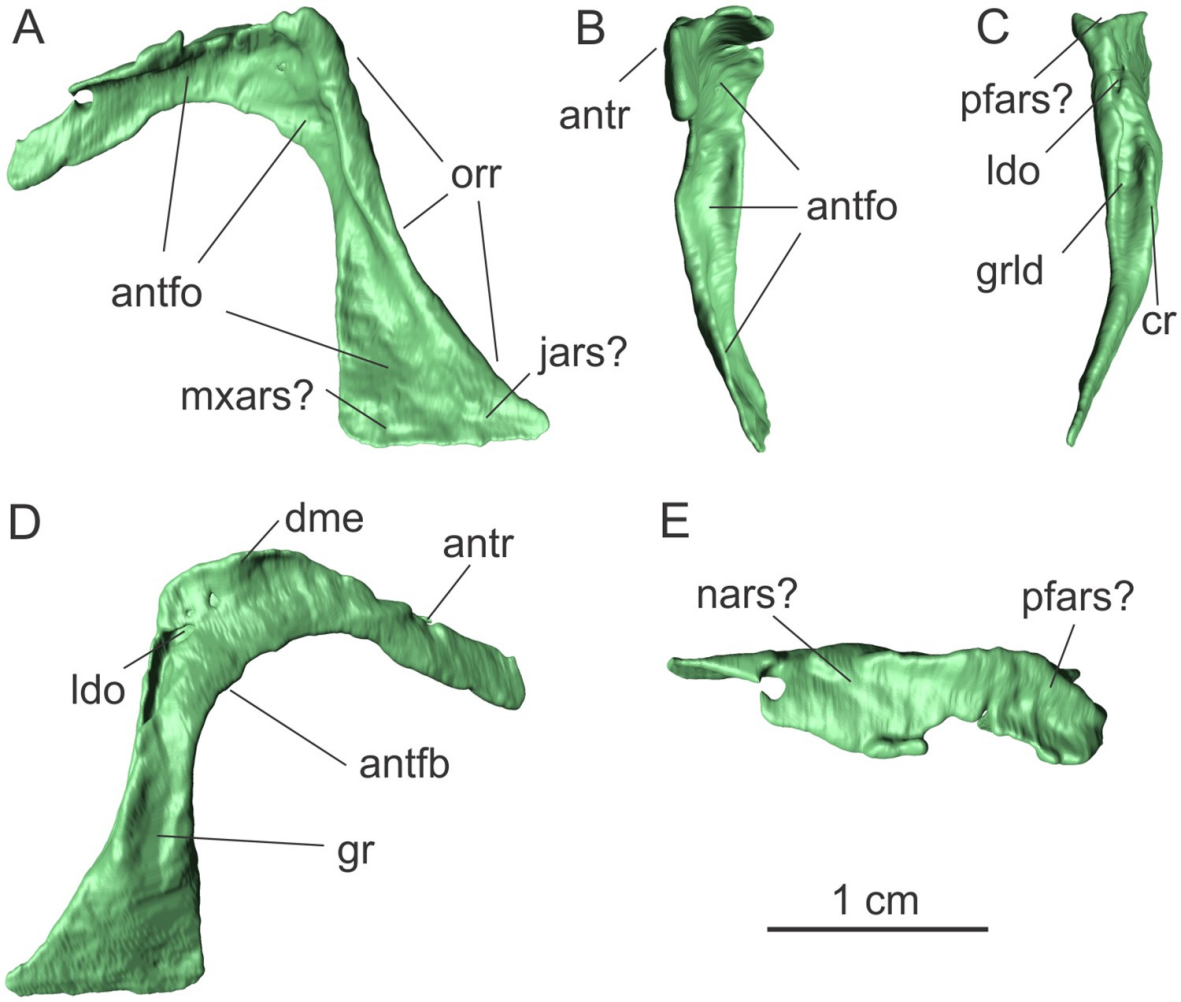
Left lacrimal of the specimen MCP-3845-PV of *Saturnalia tupiniquim* in lateral (A), anterior (B), posterior (C), medial (D), and dorsal (E) views. **Abbreviations**: **antfb—**border of the antorbital fenestra; **antfo—**antorbital fossa; **antr—**anterior ramus; **cr—**crest; **dme—**dorsomedial expansion; **gr—**groove; **grld—**groove associated with the lacrimal duct; **jars—**articulation surface with the jugal; **ldo—**lacrimal duct opening; **nars—**articulation surface with the nasal; **mxars—**articulation surface with the maxilla; **orr**—orbital rim; **pfars—**articulation surface with the prefrontal.

Left and right lacrimals are preserved in MCP-3845-PV (Fig 5). The right element is matrix-free, but incomplete, lacking the anterior ramus. Only the medial surface of left lacrimal is exposed, but the bone could be completely reconstructed using CT-Scan data. Hence, the following description is based solely on the latter element, but there are no noteworthy differences in the anatomy of the preserved parts of both lacrimals. The bone has an inverted ‘L’ shape, with a main dorsoventrally elongated body (22 mm in length), i.e. the lacrimal shaft, which marks the separation between the antorbital fossa anteriorly and the orbit posteriorly, and an anterior ramus (19 mm in length) that forms the posterior part of the dorsal margin of the antorbital fenestra. In lateral view, the main axes of these portions form a right angle at the level of the posterodorsal border of the antorbital fossa (Fig 5A).

When the ventral margin of the lacrimal shaft is horizontally aligned, the anterior ramus is anteroventrally to posterodorsally oriented. The anterior ramus can be divided in two laminar portions: a dorsoventrally compressed dorsal portion and a mediolaterally compressed ventral portion, the lateral surface of which is slightly concave dorsoventrally. Together, these portions are L-shaped in anterior view, with the dorsal portion expanding laterally (Fig 5B). However, this lateral expansion is seen only along the posterior two thirds of the anteroposterior axis of the ramus. Thus, its anteriormost portion corresponds only to a tall mediolaterally compressed lamina, which might represent the contact with the dorsal ramus of the maxilla. On the lateral surface of the lacrimal, a depression on the posterior portion of the anterior ramus, where it merges with the lacrimal shaft, corresponds to the posterodorsal corner of the antorbital fossa (‘antfo’ in Fig 5). This part of the fossa is exposed in lateral view, as also observed in *Eoraptor* and *Pampadromaeus*. The dorsal portion of the anterior ramus of the lacrimal forms a roof over the posterodorsal corner of the antorbital fossa, where the dorsal surface of the bone is transversely concave. The posteriormost region of this concavity likely represent the surface that was overlapped by the prefrontal (‘pfars’ in Fig 5E), whereas its anterior portion might represent the articulation surface with the nasal (‘nars’ in Fig 5E).

The ventral third of the lacrimal shaft is a mediolaterally compressed lamina (Fig 5). Its lateral and medial surfaces are both anteroposteriorly and dorsoventrally concave and convex, respectively. At its ventral limit, the lacrimal shaft is ca. 10 mm long anteroposteriorly, and its ventrolateral surface likely represent to articulation areas with the posterior ramus of the maxilla anteriorly (‘mxars’ in Fig 5A), and the anterior ramus of the jugal posteriorly (‘jars’ in Fig 5A). Dorsal to this laminar portion, the lacrimal shaft becomes shorter anteroposteriorly, with nearly half of the anteroposterior length of the ventral margin, but expands transversely at its posterior portion. The lacrimal shaft is not vertically straight. In posterior view, it is concave laterally and convex medially, with the change in the main axis orientation occurring exactly at the dorsal limit of the mediolaterally compressed ventral third described above, which also marks the ventral limit of a groove associated with the lacrimal duct (‘grld’ in Fig 5C). This grove extends dorsoventrally, ending dorsally in the lacrimal duct opening (‘ldo’ in Fig 5C). On the opposite side of this groove, the anterior surface of the lacrimal is transversely concave and continuous with the part of the antorbital fossa formed by the anterior expansion of the medial portion of the lacrimal shaft, which is visible in lateral view. In this view, a small part of the fossa, at the mid-length of the dorsoventral axis of the lacrimal shaft, is hidden by a sheet of bone that folds anteriorly from the lateral margin of the lacrimal.

On the medial surface of the lacrimal, a crest extends along the posterior margin of the shaft. It separates the groove associated with the lachrymal duct on the posterior surface of the bone from another dorsoventrally oriented, and anteroposteriorly concave, groove (‘gr’ in Fig 5D) on the medial surface of the bone, which extends dorsally for half the length of the posterior groove. The dorsal half of the lacrimal shaft is anteroposteriorly convex, following the concavity of the antorbital fossa on the lateral side of the bone. The medial surface of the anterior ramus also mostly follows the curvature on the opposite side of the bone, being dorsoventrally convex, except for its concave proximal third. This is due to the medial expansion (‘dme’ in Fig 5) of the dorsal surface of the anterior ramus in the region where it was likely overlapped by the prefrontal (‘pfars’ in Fig 5).

#### Postorbital

**Fig 6:**
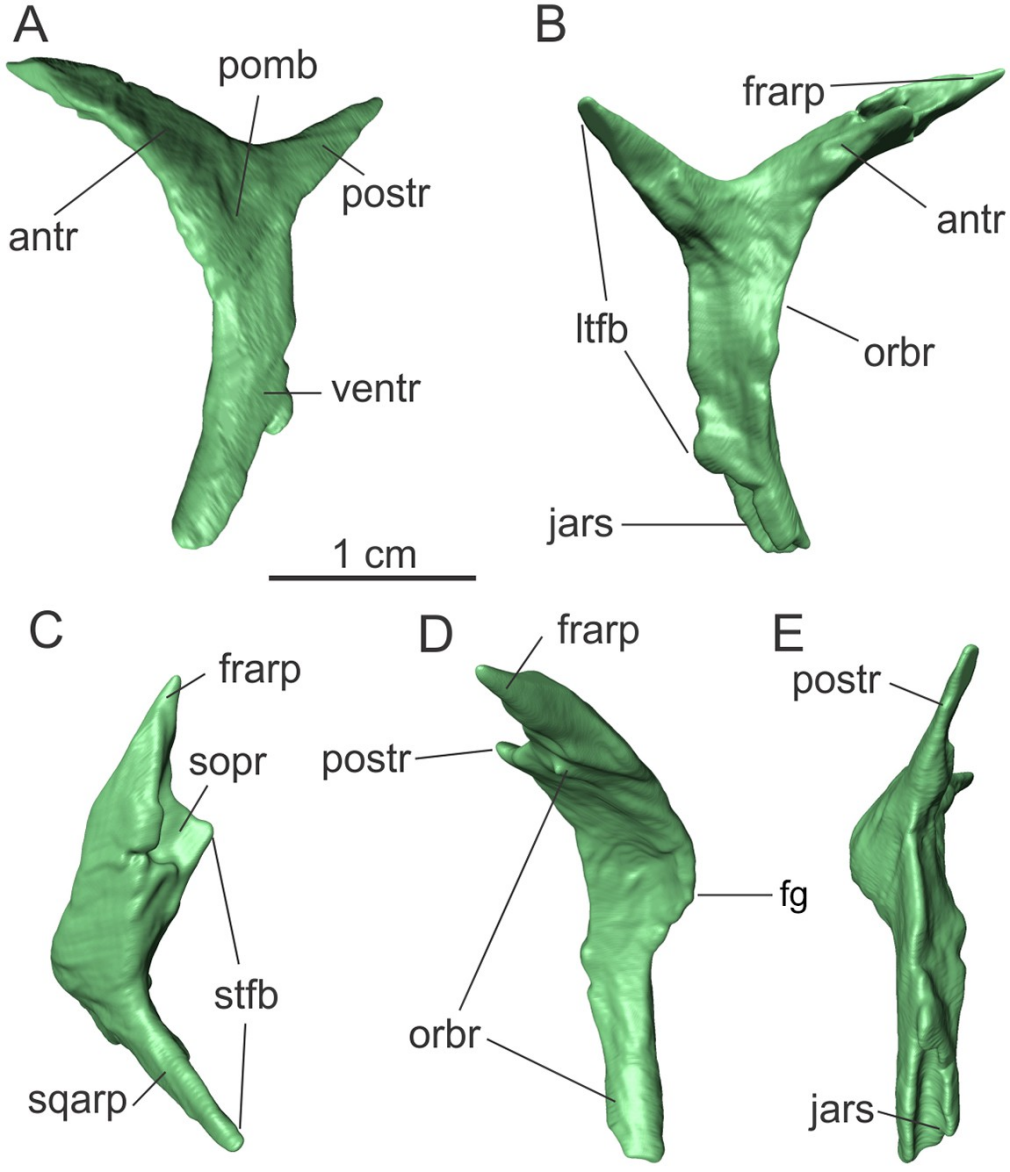
Left postorbital of the specimen MCP-3845-PV of *Saturnalia tupiniquim* in lateral (A), medial (B), dorsal (C), anterior (D) and posterior (E) views. **Abbreviations**: **antr**—anterior ramus; **fg—**flange; **frarp—**articulation process with the frontal; **jars—**articulation surface with the jugal; **ltfb—**border of the laterotemporal fenestra; **orbr—**orbital rim; **postr—**posterior ramus; **sopr—**socket for articulation with the parietal; **sqarp—**articulation process with the squamosal; **stfb—**border of the supratemporal fenestra; **ventr—**ventral ramus.

Left and right postorbitals of MCP-3845-PV are preserved (Fig 6). The left element is partially visible in the block, whereas the right element is completely hidden by matrix. The segmentation results show no differences in the morphology of the anterior and posterior rami of the left and right postorbitals. Thus, it is most likely that these rami are completely preserved in both bones. The description provided here is based solely on the left element, which possesses a more complete ventral ramus (Fig 6). The postorbital is a triradiate bone, with ventral (‘ventr’ in Fig 6), posterior (‘postr’ in Fig 6), and anterior (‘antr’ in Fig 6) rami that would have respectively contacted the jugal, the squamosal, and the frontal. In MCP-3845-PV, the ventral ramus is the longest, followed by the anterior.

The posterior ramus of the postorbital extends posteromediodorsally. In lateral view it is spike-like; wider proximally and tapering distally (Fig 6). It is also lateromedially compressed, being the thinnest of the three postorbital rami. It is nearly 9 mm long, and its distal portion would have fitted into an articulation socket located on the anterior ramus of the squamosal, together forming the dorsal margin of the lower temporal fenestra. Still in lateral view, an angle of c. 120 degrees is formed between the dorsal margins of the anterior and posterior rami of the postorbital, similar to the condition in *Buriolestes*, but different from that of *Pampadromaeus*, in which that angle is of approximately 180 degrees.

The anterior ramus is the broadest (transversally) of the three rami of the postorbital. From the centre of the bone, it projects anterodorsomedially, forming a dorsal arch, and has a length of ca. 15 mm. A socket (‘sopr’ in Fig 6) in the medial surface accommodated the lateral tip of the anterolateral ramus of the parietal (S2 Appendix). Anterolateral to this socket, a finger-like anterior process (‘frarp’ in Fig 6) tappers distally, fitting into the slot on the posterolateral corner of the dorsal surface of the frontal (S2 Appendix). The ventral surface of the anterior ramus, which formed part of the orbital rim (‘orbr’ in Fig 6), is 5 mm wide mediolaterally and concave in that same direction. At the orbital rim, where the anterior and ventral rami of the postorbital merge, the lateral margin of the bone expands anteriorly, forming a flange seen in anterior and lateral views (‘fg’ in Fig 6), similar to what is observed in *Eoraptor* (see Figure 40 in [29]) and *Buriolestes* (see Figure 9 in [26]). Ventral to this flange, the ventral ramus of the postorbital is lateromedially narrower than at the junction of the three rami, with a width of approximately 3 mm. The ventral ramus is nearly 18 mm long, but its tip is broken. The articulation area for the jugal (‘jars’ in Fig 6) is located at the posterior surface of the ventral portion of the ramus and is a lateromedially concave slot with a dorsoventral length over 7 mm. As preserved, the surface for the articulation with the jugal extends for nearly 40% of the total length of the ventral ramus.

#### Squamosal

**Fig 7:**
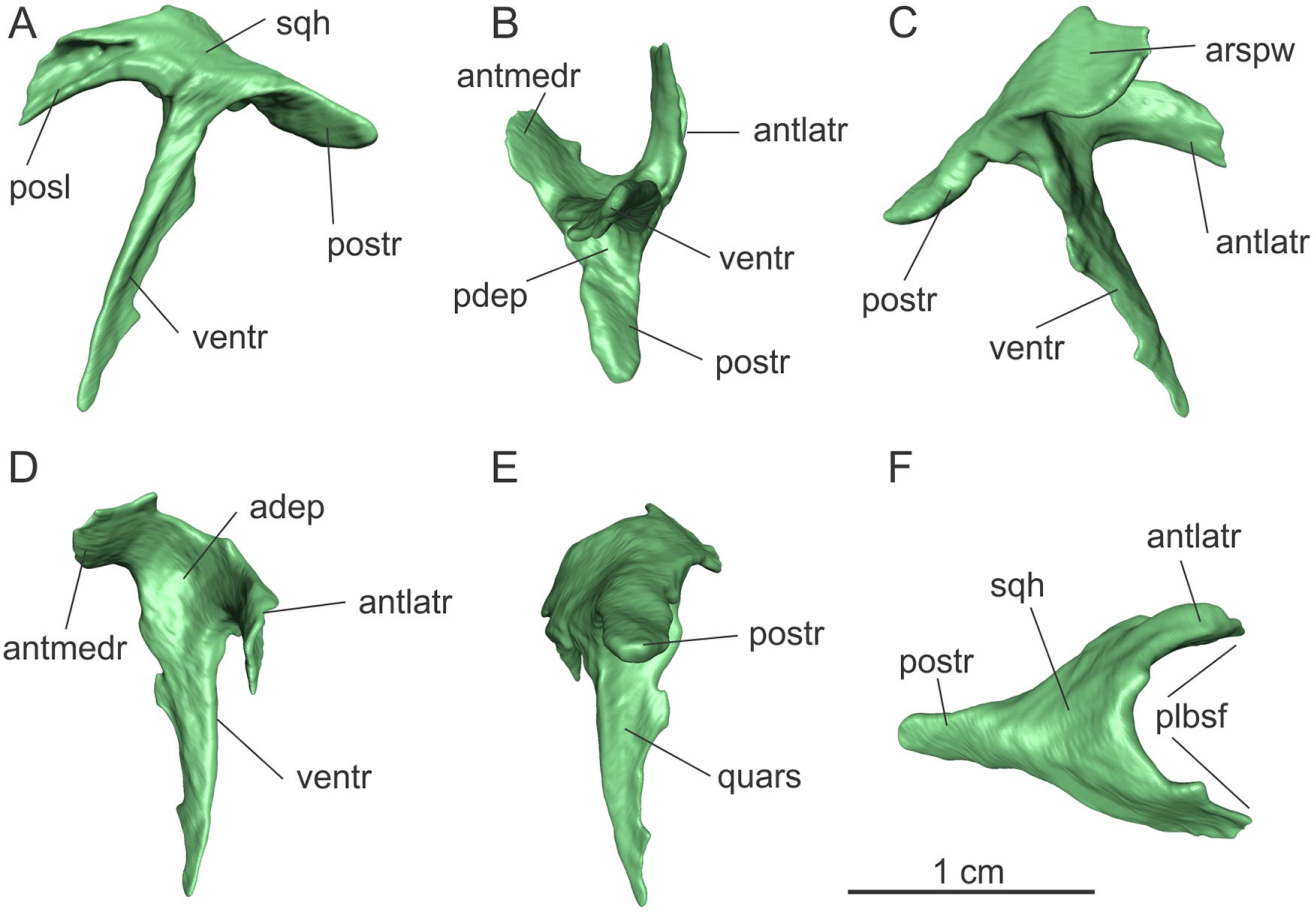
Left squamosal of the specimen MCP-3845-PV of *Saturnalia tupiniquim* in lateral (A), ventral (B), medial (C), anterior (D), posterior (E), and dorsal (F) views. **Abbreviations: adep—**anterior depression; **antlatr—**anterolateral ramus; **antmedr—**anteromedial ramus; **arspw—**articulation surface with the parietal wing; **pdep—**posterior depression; **plbsf—**posterolateral border of the supratemporal fenestra; **posl—**articulation slot with the postorbital; **postr—**posterior ramus; **quars—**articulation surface with the quadrate; **sqh—**head of the squamosal; **ventr—**ventral ramus.

Only the left squamosal of MCP-3845-PV is preserved (Fig 7). The bone is partially visible in the matrix, but additional morphological details are seen in the CT- images. The squamosal is a tetraradiate element. It appears to lack the distalmost portion of the ventral ramus, but it is otherwise completely preserved. A ventral ramus (‘ventr’ in Fig 7) formed the posterior margin of the laterotemporal fenestra and contacted the quadrate posteriorly (‘quars’ in Fig 7E). The three additional rami converge to the head of the bone, which forms the posterolaterodorsal corner of the skull. The anterolateral ramus (‘antlatr’ in Fig 7) contacted the postorbital, shaping the dorsal margin of the laterotemporal fenestra. The anteromedial ramus (‘antmedr’ in Fig 7) contacted the parietal wing and, together with the anterolateral ramus, formed the posterolateral margin of the supratemporal fenestra. Finally, the posterior ramus (‘postr’ in Fig 7) probably covered the quadrate head and contacted the paroccipital process of the otoccipital. The squamosal is over 18 mm high, from the dorsal surface of the head until the preserved tip of the ventral ramus.

The ventral ramus of the squamosal is an anterolaterally to posteromedially expanded thin lamina, but with a rounded anterior margin (Fig 7). It is nearly 14 mm high and has a maximum width of over 3 mm proximally, tapering distally. The main axis of the preserved ventral ramus is straight, as observed in *Eoraptor* and *Pampadromaeus*. As its distalmost portion is lacking, it is not possible to assert if the tip of the ramus was curved posteriorly as in *Plateosaurus* spp.

Because all the cranial elements of MCP-3845-PV are disarticulated, it is not possible to precisely establish the orientation of the squamosal. However, based on the position of the slot for the articulation with the postorbital in the anterolateral ramus, it is most likely that the ventral ramus was not vertically oriented, but would bend anteriorly at an angle of c. 30–45 degrees with the vertical axis. The posterior surface of the ventral ramus is transversally concave along its entire length, and a circular depression (‘pdep’ in Fig 7B) of nearly 3 mm in diameter is seen where this surface meets the ventral surface of the posterior ramus. The posterior ramus of the squamosal projects posterolaterally (S2 Appendix), with its main axis forming an acute angle to that of the ventral ramus in lateral view. It is finger like, with a rounded tip, slightly compressed dorsoventrally, and has a maximum length of over 7 mm. Its lateromedial width is mostly constant along its length, but its transverse long axis is inclined in a way that the lateral margin is located dorsally in relation to the medial margin.

Another depression (‘adep’ in Fig 7D) is observed where the anterior surface of the ventral ramus meets the proximal parts of the anterolateral and anteromedial rami. In dorsal view, the proximal portion of the latter two rami diverge from one another at an angle of ca. 60 degrees. Together, the rami form an arch, as given by their concave surfaces that face one another, which is the posterolateral corner of the supratemporal fenestra (S2 Appendix). The anteromedial ramus is a sharp lamina, c. 3.5 mm tall, inclined in a way that its dorsal margin is laterally displaced in relation to the ventral. Also, that ramus is ventrally curved at its distal half. It would probably have laterally overlapped the parietal wing.

The anterolateral ramus is 7.2 mm long as preserved. It bears a slot (‘posl’ in Fig 7A) for the articulation with the postorbital on its lateral surface, which corresponds to an anteroventrally to posterodorsally oriented groove (c. 6 mm long). The slot tapers posteriorly, following the shape of the distal portion of the posterior ramus of the postorbital, and almost reaches the squamosal head, but it is not visible in dorsal view. The medial surface of the anterolateral ramus chiefly follows the curvature of the respective lateral surface, being dorsoventrally convex.

#### Quadrate

**Fig 8:**
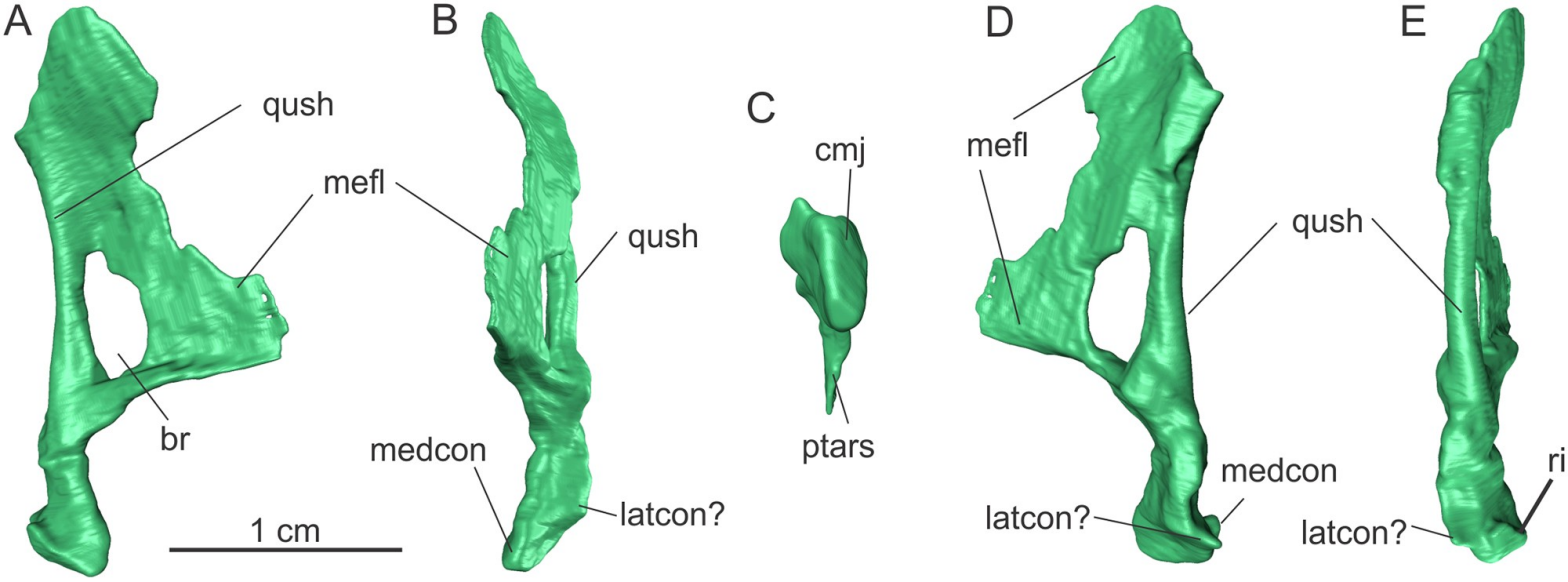
Left quadrate of the specimen MCP-3845-PV of *Saturnalia tupiniquim* in medial (A), anterior (B), ventral (C), lateral (D), and posterior (E) views. **Abbreviations**: **br—**breakage; **cmj—**craniomandibular joint; **latcon—**lateral condyle; **medcon—**medial condyle; **mefl—**medial flange; **ptars—**articulation surface with the pterygoid; **qush—**quadrate shaft; **ri—**ridge.

The specimen preserves only a partial left quadrate (Fig 8), including the quadrate shaft and the medial flange (i.e. the pterygoid ramus), and the lacking lateral flange (i.e., quadratojugal ramus). The preserved part of the quadrate shaft (‘qush’ in Fig 8) is nearly 28 mm high. Its posterior margins is gently dorsoventrally concave. The transverse width of the quadrate shaft is relatively constant along the dorsoventral axis, but it expands at its ventral fourth. This expanded area houses the condyles of the craniomandibular joint (‘cmj’ in Fig 8). The lateral condyle (‘latcon’ in Fig 8) is almost entirely absent, whereas the medial condyle (‘medcon’ in Fig 8) is better preserved. It expands ventromedially, and has a rounded ventral surface. There is a marked transverse ridge (‘ri’ in Fig 8E) on the lateral margin of the medial condyle, as seen in *Buriolestes*.

The medial flange (‘mefl’ in Fig 8) of the quadrate extends along the three dorsal fourths of the quadrate shaft. Its medial surface is concave both dorsoventrally and anteroposteriorly. This is also observed in *Panphagia*, *Pampadromaeus*, and *Plateosaurus* spp, differing from the condition of *Buriolestes*, in which that surface is anteroposteriorly convex. The ventral margin of the medial flange bends slightly dorsally as it proceeds anteriorly, forming an angle of nearly 120 degrees with the main axis of the quadrate shaft in lateral view. This margin is transversally rounded (contrasting with the more laminar dorsal portion of the flange), anteroposteriorly concave, and likely represented a contact site for the pterygoid (‘ptars’ in Fig 8C). The maximal anteroposterior extension of the medial flange (about 10 mm) is located at its ventral portion. Its dorsal portion extends less anteriorly, but the exact shape of the anterior margin is unknown due to breakage. An additional large breakage (‘br’ in Fig 8A) is seen at the contact of the medial flange with the quadrate shaft, at about the mid-dorsoventral length of the former.

#### Dentary

In the first description of *Saturnalia* [3], part of a hemimandible associated with MCP-3845-PV was briefly described as the ‘mandibular ramus’, but it was not identified as belonging to either side of the skull–mostly due to its poor preservation. Further preparation revealed the right dentary of that same specimen. Thus, the mandibular ramus described in [3] is here assigned to the left side, as also supported by its morphology, and is interpreted as composed solely of the dentary.

**Fig 9:**
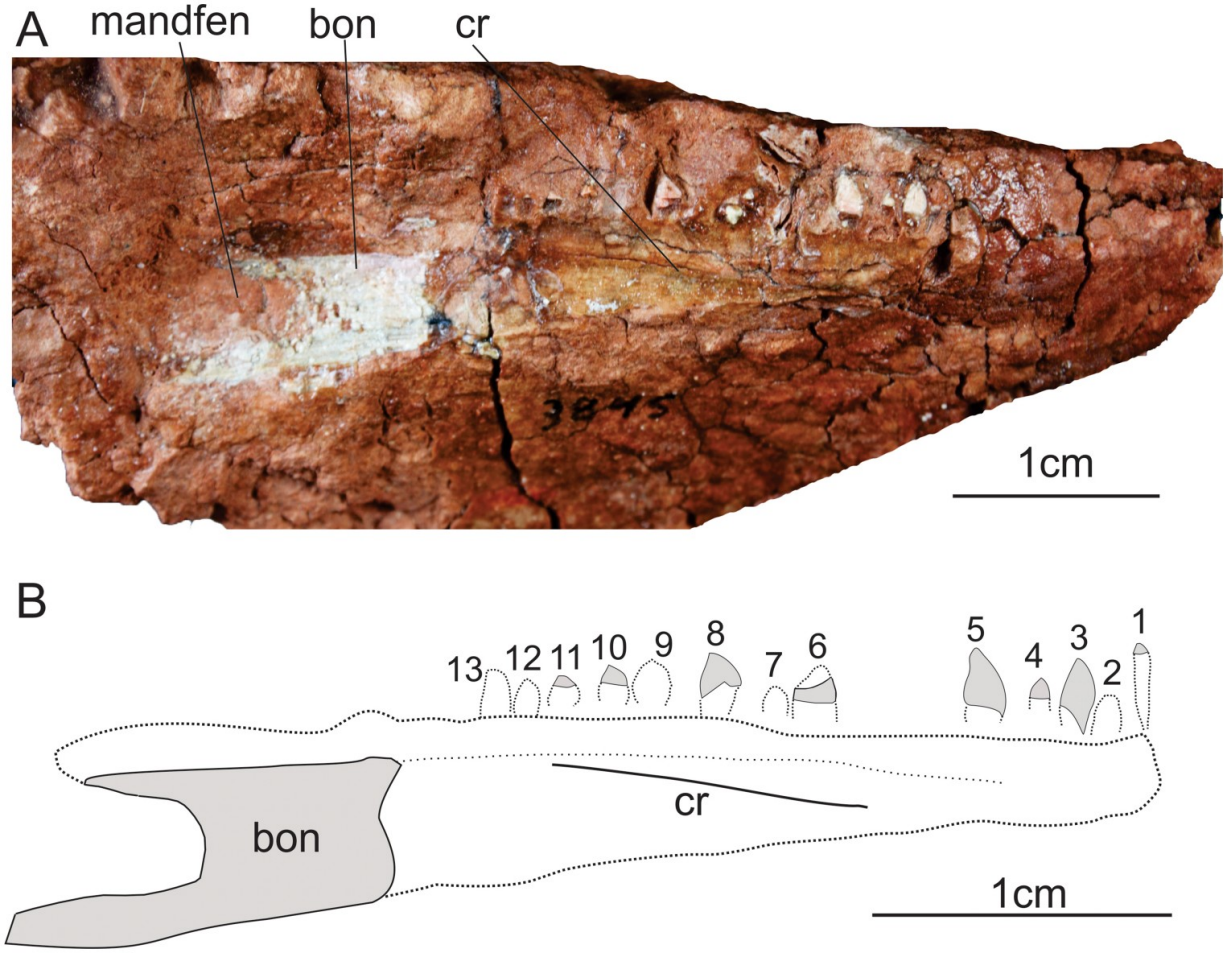
Left dentary of the specimen MCP-3845-PV of *Saturnalia tupiniquim* in medial view (A) and interpretative drawing (B). **Abbreviations**: **bon:** preserved bone; **cr—crest; mandfen—**mandibular fenestra; **tocr—**tooth crown; **1 to 13**—preserved tooth crowns.

Only the posterior portion of the left dentary (Fig 9) is preserved as bone material, with its anterior part represented by the impression left by the bone surface in the sediment. That impression bears a low “crest” extending anteroposteriorly, c. 3 mm ventral to the tooth line. This “crest” (‘cr’ in Fig 9) most likely corresponds to a groove on the lateral surface of the bone, and the presence of a groove is congruent to the morphology of sauropodomorphs such as *Eoraptor*, *Buriolestes*, *Panphagia*, and *Plateosaurus* spp. The preserved bony portion of the posterior part of the dentary is nearly 16 mm long, and an excavation on its posterior surface is interpreted as the anterior margin of the external mandibular fenestra. Thus, the left dentary seems mostly complete posteriorly. Anteriorly, its impression in the sediment extends for nearly 28 mm. However, the anterior most tooth impression is c. 33 mm ahead of the anterior limit of the preserved bone. The anterior most tooth impression (‘1’ in Fig 9) indicates that the tooth associated with it was nearly five times apicobasally longer than mesiodistally wide, with an elliptical shape in lateral view. This morphology is similar that of the first dentary tooth of *Pampadromaeus*.

**Fig 10:**
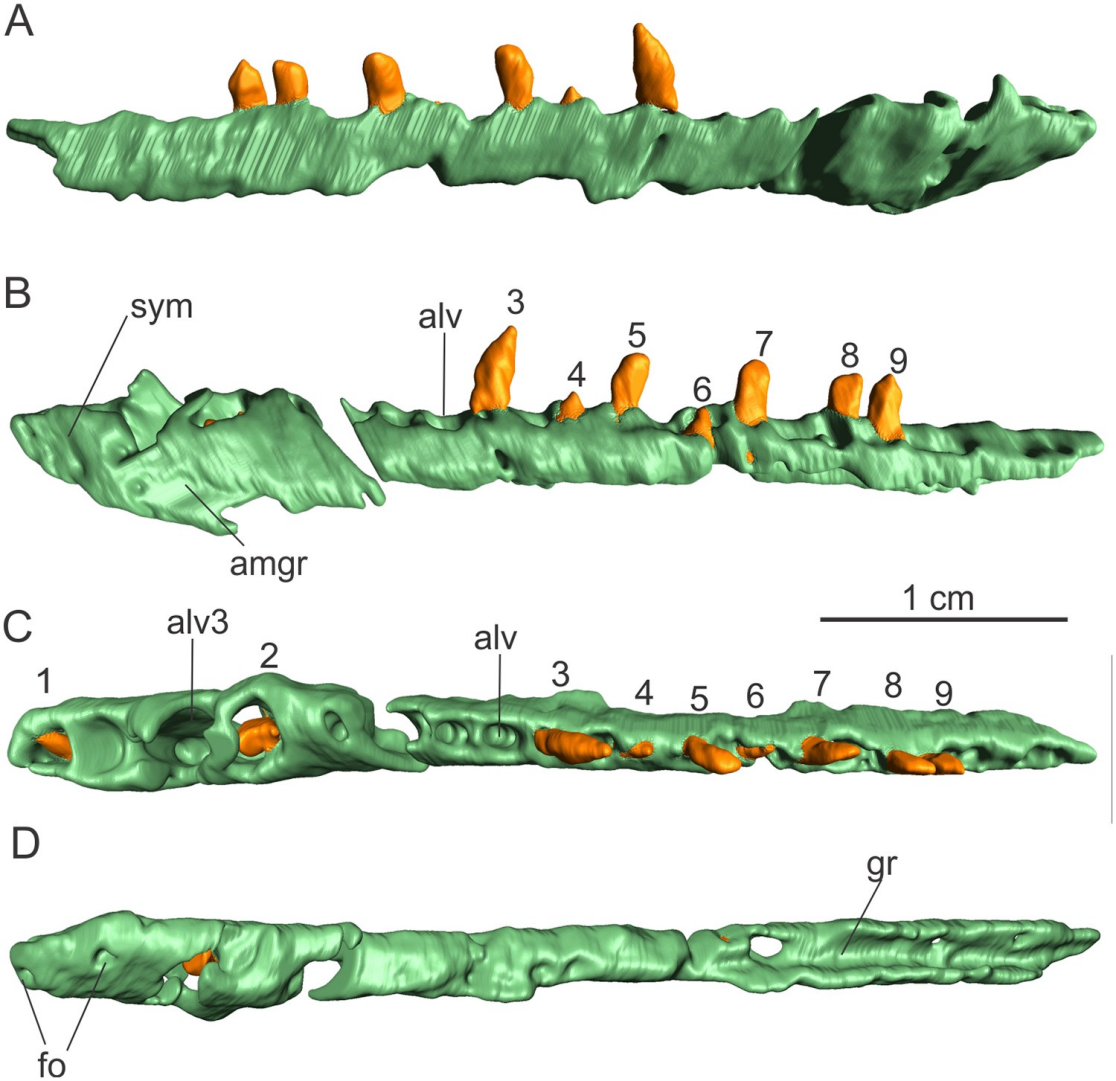
Right dentary of the specimen MCP-3845-PV of *Saturnalia tupiniquim* in lateral (A), medial (B), dorsal (C), and ventral (D) views. **Abbreviations: alv—**alveolous; **alv3 —**third alveolous; **amgr—**anteromedial groove; **fo—**foramen; **gr—**groove; **sym—**symphysis; **to—**tooth; **1 to 9**—preserved tooth crowns.

The right dentary (Fig 10) is more complete than the left, but it lacks all its ventral margin posterior to the symphyseal region. It is also broken at the level of the 5^th^ alveolus, being thus preserved in two separated pieces. The posterior piece is mostly straight in dorsal/ventral views. Most of the dentary surface, especially the lateral side, is hidden by matrix, but it could be reconstructed using CT-Scan data. We could not identify an anteroposteriorly oriented groove on the lateral surface of the bone, as inferred for the left element. However, this absence can be an artefact of the CT data segmentation, lack of the portion of the lateral surface bearing the groove (see Fig 9), or even a preservation problem. For instance, such a groove is clearly seen on the left dentary, but not in the right dentary of the specimen AMNH 6810 of *Plateosaurus erlenbergensis* (see e.g. Figures 31 and 32 in [17]).

The anteroposterior length of the right dentary as preserved is nearly 46 mm, with a total of 21 to 22 tooth positions, a number that is mostly equivalent to seen in other early sauropodomorphs (e.g. *Eoraptor–* 20 dentary teeth; *Pampadromaeus–*minimum of 18 dentary teeth; *Plateosaurus* spp*–*ca. 22 dentary teeth). The most posterior of the preserved alveoli is located almost at the posterior edge of the bone. Thus, based on comparisons with the tooth number and position of other sauropodomorphs such as *Panphagia* and *Plateosaurus erlenbergensis* (AMNH 6810) the right dentary of MCP-3845-PV was likely slightly longer than preserved. Eight teeth have completely or partially preserved crowns and one (4^th^) has only part of its root preserved inside the alveolus (Fig 10). The second, third, and fourth alveoli are two to three times larger than the others and the first four alveoli have a sub-circular shape, differing from the more elliptical posterior alveoli, which are slightly anteroposteriorly longer than wide.

There is no evidence of the presence of a predentary bone at the anterior portion of the dentary. The symphysis is short, only reaching the level of the third alveolus (‘alv3’ in Fig 10), and two foramina (‘fo’ in Fig 10) pierce the bone surface ventral to that. The anterior foramen is at the anteroposterior level of the first alveolus and more dorsally located in relation to the posterior, which is at the level of the second alveolus. Additionally, a large number of smaller unevenly distributed pits are present along the lateral surface of the symphyseal area, but these cannot be recognised in the digital model of the dentary. The alveolar margin of the symphyseal portion of the dentary slopes slightly ventrally as it proceeds anteriorly. Posterior to that, the alveolar margin is straight. The first dentary tooth has the tip of its crown exposed (‘1st’ in Fig 10), but this is not visible in lateral view, indicating that it was still not fully erupted. Nevertheless, the anterior border of the first alveolus is located at the anterior extremity of the dentary, and it is not broader than those of the other alveoli. Thus, as in *Pampadromaeus* and *Buriolestes*, there is no gap between the first tooth position and the anterior tip of the dentary, differing from the condition inferred for *Eoraptor* [29] and *Panphagia*, and more clearly observed in later sauropodomorphs such as *Plateosaurus* spp and *Massospondylus* spp. In lateral view, the anterior tip of the dentary has a triangular shape, pointing forwards (Fig 10). This morphology is more similar to that of *Buriolestes* and *Eoraptor*, differing from that of *Plateosaurus* spp, which possesses a more rounded anterior margin of the dentary in lateral view.

In ventral view, it is possible to observe an anteroposteriorly oriented groove (‘gr’ in Fig 10D) extending between the lateral and medial margins of the dentary. This likely represents the posterior portion of the meckelian channel, which must have been also enclosed medially by the splenial and angular. Anteriorly, adjacent to the posterior end of the symphysis, a posteroventrally to anterodorsally oriented groove (‘amgr’ in Fig 10B), nearly 4 mm long, is visible in medial view, which might represent the anterior portion of the meckelian groove. However, the ventral surface of the dentary is damaged anteriorly, in a way that it is not possible to safely infer the total anterior extension of the groove. There is no visible connection between the grooves at the anterior end of the dentary and on the ventral surface of its posterior portion, but that might be due to the bad preservation of the ventral portion of the bone.

#### Dentition

For the description, teeth were labelled (Figs 1, 9 and 10) according to their relative position along the antero-posterior axis (i.e. anterior most preserved tooth was labelled as ‘1’) and not according to the position they would occupy in the tooth row of the living animal.

**Fig 11:**
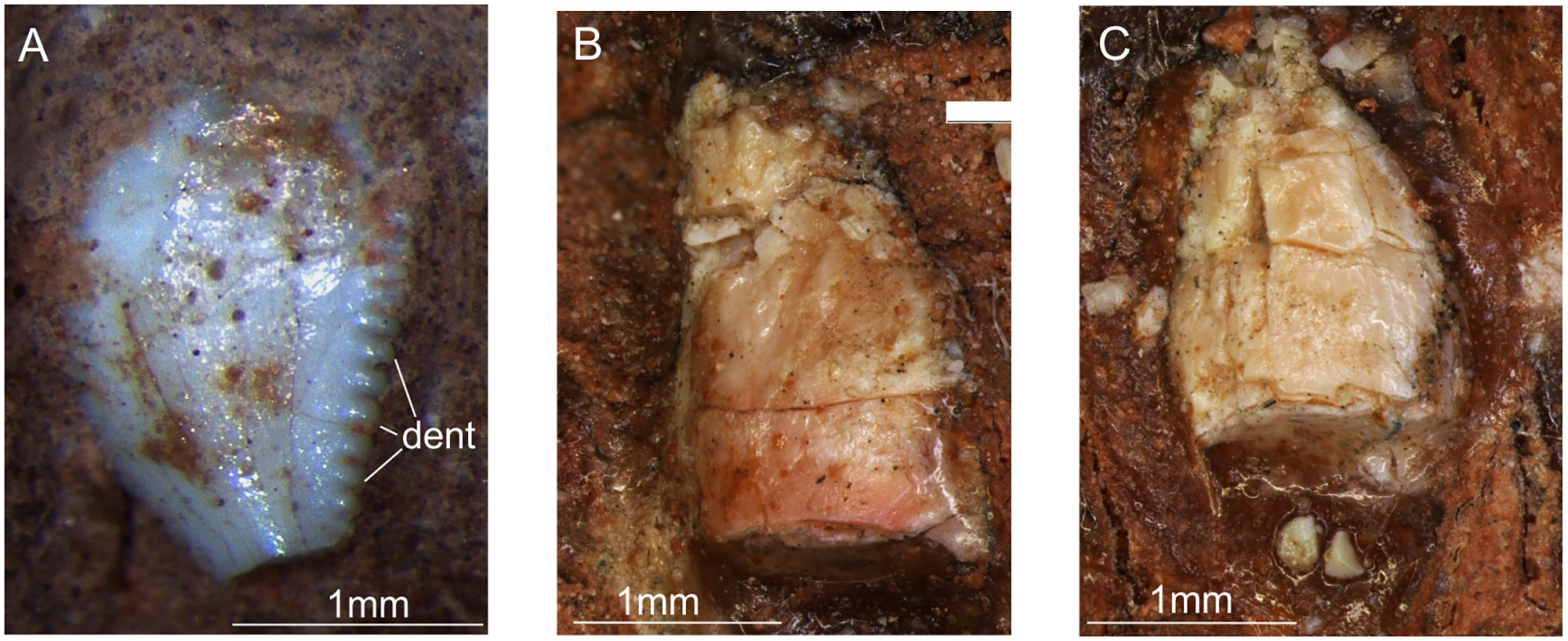
Dentition of *Saturnalia tupiniquim*. A. Sixth (from anterior to posterior) of the preserved teeth in the left maxilla of the specimen MCP-3845-PV of *Saturnalia tupiniquim* in labial view. B and C. Fifth and third (from anterior to posterior) of the preserved teeth in the left dentary of the specimen MCP-3845-PV of *Saturnalia tupiniquim* in lingual view. Abbreviations: dent—denticles.

Eight teeth are preserved in the left maxilla (‘1–8’ in Fig 1). Of these, only one has an almost complete crown, which is exposed in labial view, whereas the other seven have only the root and the base of the crown preserved. The crown of the more complete tooth (‘6’ in Figs 1 and 11A) lacks its tip and also part of the base of its mesial margin. Nevertheless, it is possible to infer that it was more anteroposteriorly expanded at the base than distally. The crown is expanded labiolingually at its centre, becoming progressively thinner mesially and distally. Denticles were preserved only on the distal margin, and are mostly perpendicular to the tooth margin. There are 9–10 denticles per millimetre, as in *Buriolestes*, but differing from the coarser denticles of *Plateosaurus* spp and *Bagualosaurus agudoensis*. The apicobasal length of the crown is nearly 3.3 mm.

Nine teeth are partially preserved in the right dentary (‘1–9’ in Fig 10), but only one (‘3’ in Fig 10) has a relatively complete crown, whereas other teeth have only the base of the crown preserved (Fig 10). Tooth ‘3’ in Fig 10, which occupies the 8^th^-9^th^ alveolus has an apicobasal length of nearly 3.5 mm and a convex mesial margin and a straighter distal margin, resulting in a crown slightly curved posteriorly. The base of the crown is not preserved, but its impression on the sediment indicates that it was constricted. Other crowns are poorly preserved in a way that it is not possible to infer their medial and distal outlines.

The left dentary exhibits eight fragmentary and poorly preserved tooth crowns. However, the impression of these and of another five tooth crowns are visible in their bearing matrix, in a way that the shape and size of the crowns (‘1–13’ in Fig 9) can be approximately inferred. The most anterior of the impressions (‘1’ in Fig 9) shows that the tooth in this position was apicobasally elongated and mesiodistally narrow, as is the case of the first dentary tooth of *Pampadromaeus*. Tooth ‘5’ in Fig 9 (also Fig 11B) is slightly curved posteriorly, with convex mesial and sigmoid distal margins. Teeth ‘1’ to ‘5’ (Fig 9) have very different sizes, with teeth ‘1’, ‘3’ (Fig 11C), and ‘5’ displaying an apicobasal length twice those of teeth ‘2’ and ‘4’. The crowns of teeth ‘6’ to ‘12’ (Fig 9) are convex both mesially and distally, and their impressions (especially those of teeth ‘8’ to ‘12’) indicates that they could be constricted at the base; hence, mostly matching a leaf-shape morphology.

### Skull length of *Saturnalia tupiniquim*

The linear regression employed to estimate the cranial length of *Saturnalia* (Table 1) provided a value (R^2^ = 0.95) between 89.5 mm (minimum value obtained based on the length of the frontal as preserved) and 103 mm (maximal value, with the addition of 15%). The calculations for the mandible provided minimum and maximal values (R^2^ = 0.99) of 79.6 mm and 101.9 mm, respectively, based on the anteroposterior length of the dentary (Table 1). Thus, the estimates for the cranium and the dentary are compatible to one another.

**Table 1:** Frontal, skull, dentary, and mandible lengths of Late Triassic and Early Jurassic dinosaur taxa, and the archosauriform Euparkeria. Skull and mandible lengths for the specimen MCP-3845-PV of *Saturnalia tupiniquim* correspond to the values obtained using linear regression based on the values for the other taxa listed (see details in the Material & methods section).

TAXON	SPECIMEN	FRONTAL	SKULL	DENTARY	MANDIBLE
*Saturnalia tupiniquim*	MCP 3845 PV	29–33	89.5–103	44–59.3	79.6–101.9
*Adeopapposaurus mognai*	PVSJ 610; PVSJ 568	47	160	87	166
*Buriolestes schultzi*	CAPPA/UFSM 0035	38	108	65	111
*Coelophysis bauri*	Cast of AMNH FR 7224			144	215
*Efraasia minor*	SMNS 12668			113	160
*Eocursor parvus*	SAM-PK-K8025			43	73
*Eoraptor lunensis*	PVSJ 512	36	123	85	110
*Euparkeria capensis*	SAM-PK-K5867	32	98	55	90
*Herrerasaurus ischigualastensis*	PVSJ 407	85	300	155	280
*Heterodontosaurus tucki*	SAM-PK-K-1332	40	127	75	120
*Massospondylus carinatus*	SAM-PK-K-1314	47	159	87	139
*Panphagia protos*	PVSJ 874			75	121
*Plateosaurus erlenbergensis*	AMNH 6810	104	330	177	326
*Riojasaurus incertus*	PULR 056	76	250	160	240
*Zupaysaurus rougieri*	PULR 076	140	490	330	470

### Phylogenetic analyses

**Fig 12:**
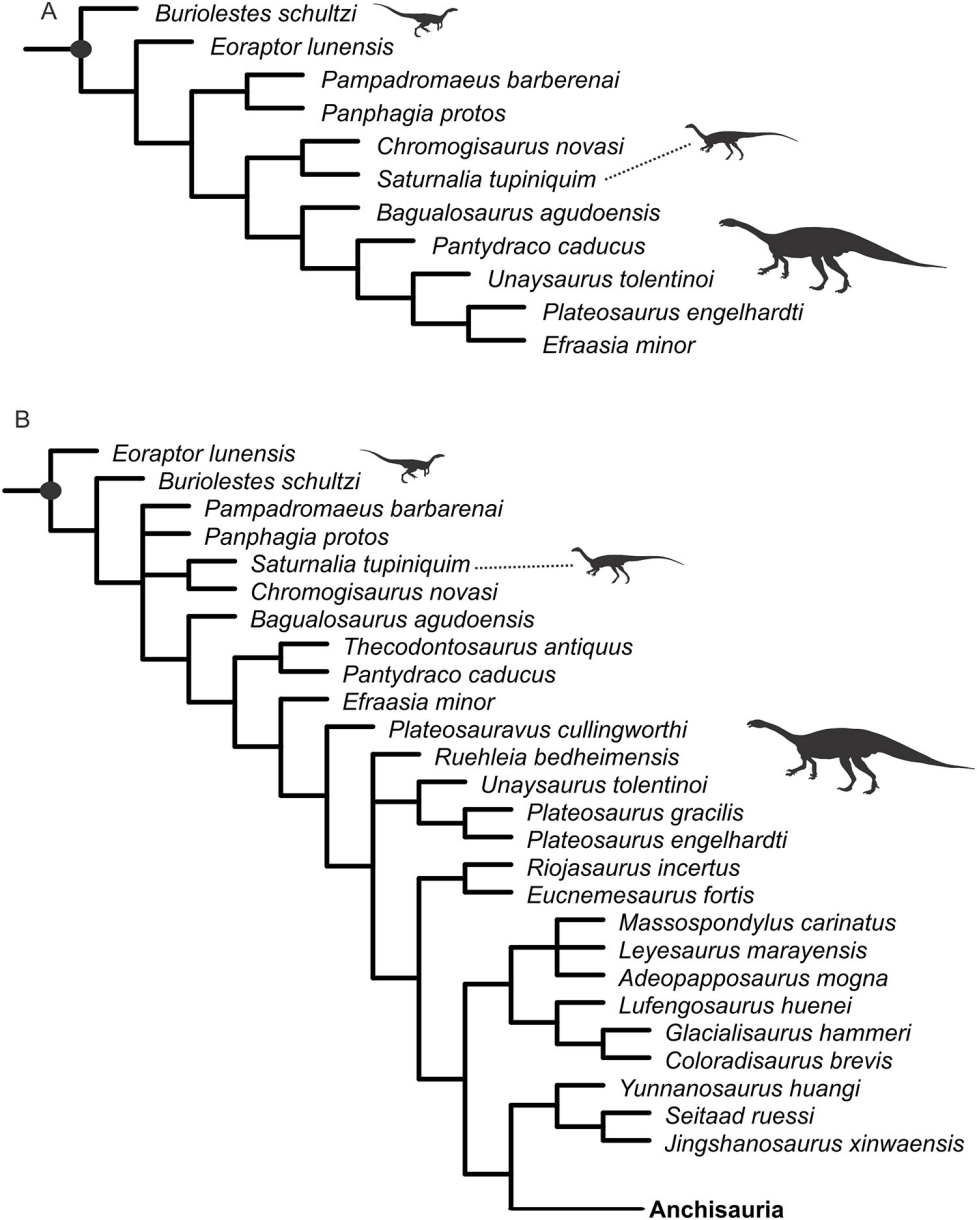
Results of phylogenetic analyses. **A.** Strict consensus trees of the 32 MPTs recovered in the phylogenetic analysis focusing on early dinosaur taxa. B. Strict consensus of the 150 MPTs recovered in the phylogenetic analysis focusing on non-neosauropodan sauropodomorphs.

The strict consensus of the 32 MPTs obtained in the analysis of the modified version of the data matrix of [8] is equivalent to that recovered in the original study as for taxa outside Sauropodomorpha (Fig 12A). Within this clade, *Buriolestes* was also recovered as the sister-taxon of all other sauropodomorphs. However, differently from the results in [8], *Pampadromaeus* and *Panphagia* form a clade that is more closely related to taxa such as *Saturnalia* and Norian sauropodomorphs than to *Buriolestes* and *Eoraptor*. As in [8], *Saturnalia* and *Chromogisaurus novasi* are recovered as sister-taxa, here more closely related to taxa such as *Bagualosaurus* (not included in [8]) and *Plateosaurus* than to the other Carnian sauropodomorphs (Fig 12A).

The phylogenetic analysis using a modified version of the dataset of [18] recovered a total of 150 MPTs. Their strict consensus (Fig 12B) differs from the results of the previous analysis (Fig 12A) in the position of some Carnian taxa. *Eoraptor*, instead of *Buriolestes*, was recovered as the sister-taxon of all other sauropodomorphs. The *Saturnalia tupiniquim* and *Chromogisaurus* clade is recovered in a polytomy with *Pampadromaeus*, *Panphagia*, and a large clade composed by *Bagualosaurus* and post-Carnian sauropodomorphs (Fig 12B).

### Principal co-ordinates analysis

**Fig 13:**
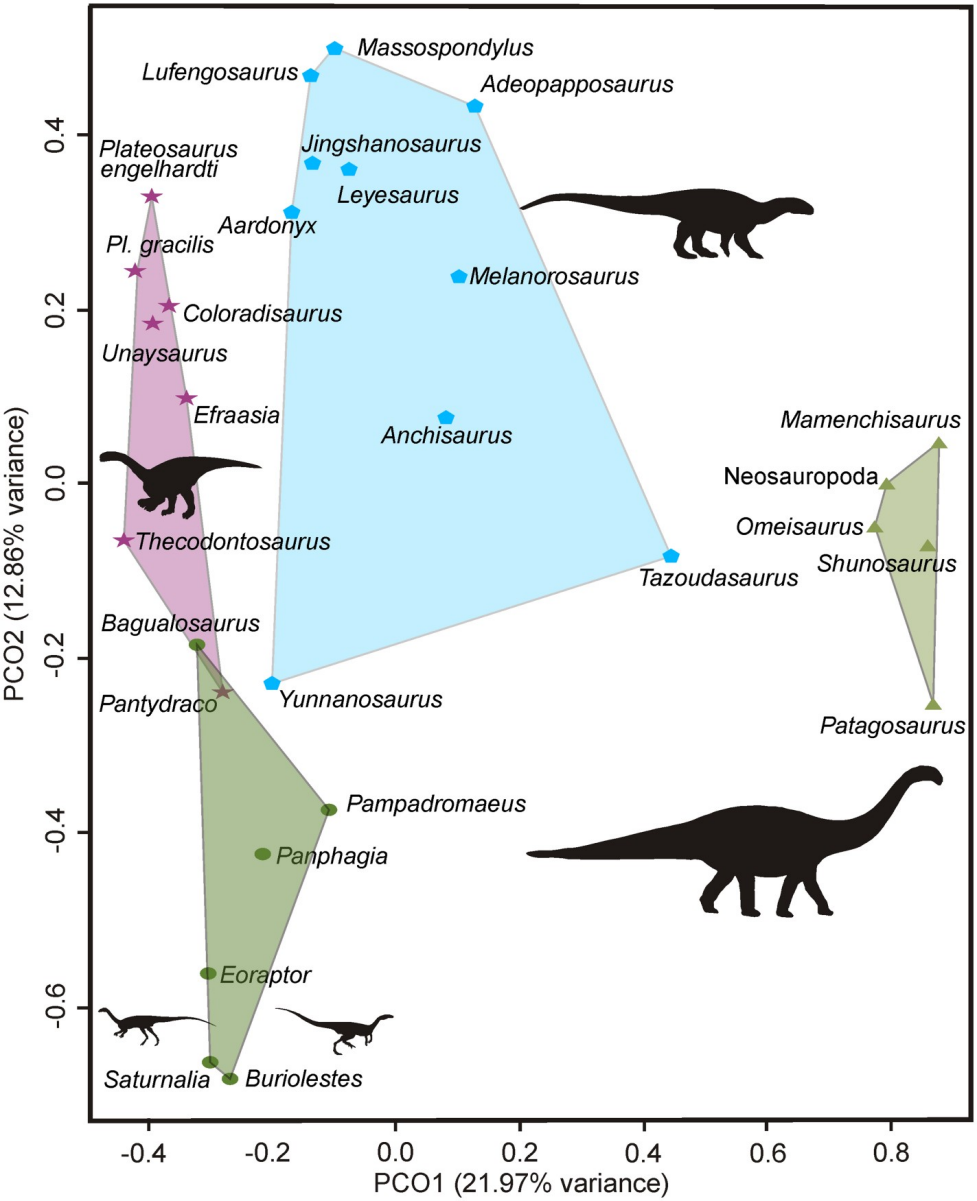
Results of the principal coordinate analysis based on cranial characters associated with the jaw feeding apparatus of sauropodomorphs with the variation in PCo1 plotted against PCo2. Circles, stars, pentagons, and, triangles, are assigned to taxa from the respective time intervals: Carnian (Late Triassic), Norian-Rhaetian (Late Triassic), Early Jurassic, and, Middle Jurassic.

**Fig 14:**
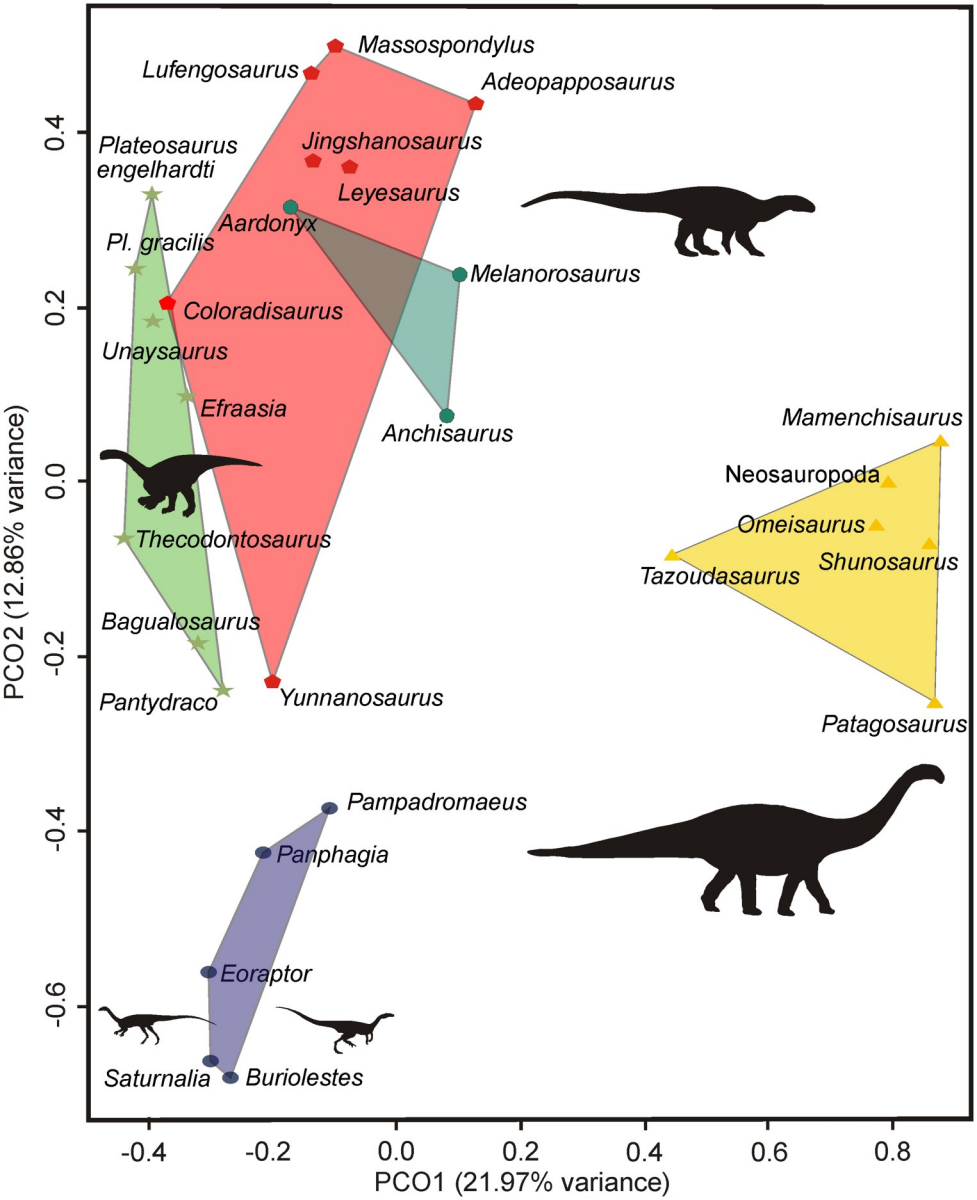
Results of the principal coordinate analysis based on cranial characters associated with the jaw feeding apparatus of sauropodomorphs with the variation in PCo1 plotted against PCo2. Circles, stars, pentagons, octagons, and, triangles, are assigned to the following subset of taxa, respectively: sauropodomorphs more closely related to *Saturnalia tupiniquim* than to *Bagualosaurus agudoensis*; non-massopodan sauropodomorphs (except those represented by circles); non-anchisaurian massopodans; non-sauropodan anchisaurians; sauropods.

The results of our PCoA analysis show that PCo1 and PCo2 together account for 34.83% of the variation in the jaw feeding apparatus character distance matrix (Figs 13 and 14). When PCo1 is plotted against PCo2, it is possible to see that the jaw feeding apparatuses of Triassic taxa occupy a region of the morphospace different from that occupied by that of Jurassic taxa, with no overlap between them (Fig 13). This is, however, not the case when PCo1 is plotted against PCo3 (the latter accounting for 6.34% of variation) nor when plotting PCo2 against PCo3 (but see Discussion below). In addition, the separation of the jaw feeding apparatus morphospace occupied by small Carnian sauropodomorphs (i.e. *Buriolestes*, *Pampadromaeus*, *Panphagia*, and *Saturnalia*) and by other sauropodomorphs is evident when plotting PCo1 against PCo2 (Fig 14) and PCo2 against PCo3 (S3 Appendix).

When morphospace occupation is defined based on groups of taxa according to their age, the area occupied by Early Jurassic taxa (which includes the largest number of taxa) is greater than that occupied by taxa from other time intervals (Fig 13). On the other hand, when morphospace occupation is investigated based on groups defined according to their phylogenetic position, that occupied by non-anchisaurian massopods shows the greatest range compared to the other groups defined here (Fig 14).

## Discussion

### Dentaries and skull size

The element here identified as the left dentary (Fig 9) has an anteroposterior length of nearly 44 mm from the tip of the anterior most tooth impression until the anterior margin of the external mandibular fenestra. This value nearly matches the anteroposterior length (c. 44.5 mm) of the tooth row in the element here identified as the right dentary of MCP-3845-PV (Fig 10). Thus, if the tooth bearing area of both dentaries are similar in length, and considering the anterior most preserved impression in the left element as that of its original first tooth, the tooth row would extend slightly posterior to the anterior margin of the external mandibular fenestra in the left dentary. Nevertheless, a most likely scenario is that the morphology of *Saturnalia* would be more similar to that observed in taxa such as *Pampadromaeus*, *Panphagia*, *Plateosaurus*. spp., and the saurischian *Tawa hallae¸* with the tooth row not extending posteriorly beyond the anterior margin of the mandibular fenestra.

The ratio ‘anteroposterior length of the tooth row/length from the tip of the dentary anteriorly till the anterior margin of the external mandibular fenestra posteriorly’ corresponds to nearly 0.75 in *Panphagia*, 0.8 in *Plateosaurus erlenbergensis* (AMNH 6810), and 0.85 in *Pampadromaeus* and *Tawa*. In this case, if we assume that the condition of *Saturnalia* matches that of *Panphagia* (which has the comparatively shorter tooth row), the length of the region between the anterior tip of the dentary and the anterior margin of the external mandible fenestra in would be about 59.3 mm in the right element of MCP-3845-PV of *Saturnalia*. In this case, the anteroposterior length of this region of the right dentary would be nearly 15 mm longer than in the left element as preserved. This discrepancy could imply that left and right dentaries referred to MCP-3845-PV did not belong to the same individual. However, such discrepancy decreases if we assume that the condition in *Saturnalia* was more similar to that of *Pampadromaeus*. In this case, the length from the anterior margin of the dentary until the anterior margin of the fenestra of the right dentary would be nearly 52.35 mm; only c. 8 mm longer than the left element. It is, however, also necessary to consider the possibility that the anterior most preserved tooth impression of the left dentary might not correspond to the original first tooth—an inference made partially based on the similarity observed between this impression and the first dentary tooth of *Pampadromaeus*. In this sense, the first impression left on the sediment associated with the left dentary of MCP-3845-PV could correspond to a more posterior tooth. Hence, the left element might have been somewhat longer, also decreasing the size discrepancy between left and right dentaries.

In sum, our estimates for the anteroposterior length of each dentary of MCP-3845-PV slightly differ, providing evidence against the assumption that they belonged to the same individual. However, such length estimates were made based on fragmentary bones without directly comparable parts. In this way, the fact both elements were found in association with the postcranial skeleton of a single individual might be a stronger evidence supporting their referral to the same specimen than the above slightly different length estimates.

Regardless of the association of the preserved dentaries to the same individual, mandible length estimates based on the minimal (44 mm) and maximal (60 mm) values for the portion of the dentary discussed above indicate minimal and maximal anteroposterior lengths for the mandible as 79.6 and 101.9 millimetres. These values are congruent with the minimal and maximal values of cranial length estimated based on the size of the right frontal, 89,5 and 103 millimetres. Thus, all values recovered in our estimates indicate that the skull length of *Saturnalia* accounts for less than two thirds of its femoral length; which is 156 mm for MCP-3845-PV [15].

### Skull reduction in sauropodomorpha

Langer et al. [3] originally estimated the skull of *Saturnalia* to be ca. 10 cm, based on the size of the left dentary of MCP-3845-PV, but no detailed measurements or calculations were provided. Our results (see above) agree with that overall estimate by [3]. A reduced skull (i.e. anteroposterior length corresponding to less than two thirds of the femoral length) is typical of sauropodomorph taxa within the less inclusive clade containing *Plateosaurus* and sauropods [4,5,11,18,30]. As our estimates indicate that this condition was also present in *Saturnalia*, there are several alternative scenarios to explain the pattern of skull reduction within Sauropodomorpha, which are related to different phylogenetic arrangements for the Carnian taxa. In some phylogenetic hypotheses (e.g. [18, 26, 31]), *Saturnalia* is found more closely related to Carnian forms that lack the reduced skull (e.g. *Buriolestes*, *Eoraptor*, *Pampadromaeus*, *Panphagia*) than to later forms such as *Plateosaurus* and sauropods. In this case, the reduced skull of *Saturnalia* would correspond to an independent acquisition relative to that observed in the later taxa. This is also a possible scenario under one of the phylogenetic arrangements presented here (Fig 12B), in which the *Saturnalia* + *Chromogisaurus* clade is recovered in a polytomy (Fig 12B). A different pattern emerges from phylogenetic hypotheses where *Saturnalia*, or the clade it forms with *Chromogisaurus* (for which no cranial remains are known), is found more closely related to later forms than to other Carnian taxa (e.g. [4,5,26,32]); as seen in Fig 12A. In this case, a single event at that clade including *Saturnalia* and such latter forms is required to explain the skull reduction in the lineage.

The short skull is a well-recognized sauropodomorph feature, but the driving force behind this anatomical modification still remains enigmatic. The only offered explanation was that a small skull on a long neck might have allowed an omnivorous animal to secure small prey items by rapid head and neck movements [33]. If the reduction of the skull only happened once, at the least inclusive clade including *Saturnalia* and later sauropodomorphs, this idea gains support from both tooth and braincase anatomy of MCP-3845-PV. Some of its mandibular teeth are posteriorly curved and a middle maxillary tooth has small serrations set perpendicular to the tooth margin. This differs from the oblique coarse serrations that are correlated to more herbivorous diets [34], departing from an herbivorous or even omnivorous diet. Regarding neuroanatomical evidences, *Saturnalia* exhibits an enlarged part of the cerebellum associated with the floccular fossae lobe (sensu [35]). Large tissue volumes (i.e. flocculus and paraflocculus) in this region of the brain would have increased gaze stability and the ability to coordinate head and neck movements [36–38], allowing a more effective predatory behaviour. For instance, predatory birds usually show higher volumes of these tissues in comparison to their non-predatory relatives [35]. Given its small body size and reduced head, *Saturnalia* was likely unable to prey on medium/large tetrapods of the time. However, a small, light skull on a relatively elongated neck could have allowed the rapid head movements require to pursuit small, elusive prey items, such as insects and small vertebrates [38].

In the broader context of sauropodomorph evolution, the specialization inferred for *Saturnalia* can highlight an important aspect of the evolutionary process, exaptation [39]. It has been demonstrated that a series of evolutionary innovations were crucial for the high efficiency of the strictly herbivore lifestyle of sauropods [40]. Among these, skull reduction significantly reduced the biomechanical constraints for neck elongation [41]. In turn, an elongated neck allowed access to food resources that were unreachable for other herbivores and created a larger feeding envelope, reducing energy consumption during food intake [42]. Thus, the idea that skull reduction was first acquired in a likely predatory member of the sauropodomorph lineage (i.e. *Saturnalia*) implies a scenario where a trait related to one habit (faunivory) was crucial for the evolution of a completely different lifestyle (herbivory) in a subsequently different selection regime.

### Early evolution of the jaw feeding apparatus of sauropodomorphs

Based on recent fossil discoveries and reassessments of early dinosaur phylogeny, it is possible to trace a more detailed scenario for the evolution of the feeding behaviour of sauropodomorphs during the first 30 million years of their evolutionary history (see also [43,44]). A recently described taxon from the Late Triassic (Carnian–c. 230 Ma) Santa Maria Formation of Brazil, *Buriolestes* [8,26], possesses a typically carnivorous tooth morphology, i.e. posteriorly curved teeth, knife-like serrations on mesial and distal carinae, no overlap between tooth crowns [8,26]. Hence, if *Buriolestes* is the sister group of all other sauropodomorphs, as recovered in one of the analysis presented here (Fig 12A), as well as in [8] and some of the analyses of [26], the most parsimonious scenario is that its faunivory corresponds to the retention of the ancestral diet for Saurischia [34,45], or even Dinosauria [8]. Yet, if *Buriolestes* is not the sister-group of all other sauropodomorphs (Fig 12B), its diet would have to be interpreted as a reversal to the saurischian plesiomorphic condition.

The phylogenetic position of the Carnian dinosaur *Eoraptor* is still on dispute [46], with analyses placing it as a member of either Theropoda [34,47] or Sauropodomorpha [8,26]. Recently, in a reassessment of its anatomy [29], Sereno et al. [29] stated that most aspects of its tooth morphology, such as the presence of a first dentary tooth offset from the anterior end of the mandible and suppression of crown curvature, indicate a partially, or even fully herbivorous diet. Yet, the former feature has been demonstrated not to be significantly correlated to the acquisition of an herbivorous diet in dinosaurs [34], and if curvature is indeed absent in some *Eoraptor* teeth, it is not in all of them (pers. obs.). Thus, the diet assessment of *Eoraptor* is still inconclusive. Similarly, *Pampadromaeus* bears traits that hampers the recognition of a fully herbivorous or faunivorous feeding behaviour. For instance, the fourth premaxillary tooth has serrations at both, mesial and distal carinae, a trait that has so far been recognized only in *Buriolestes* among Carnian sauropodomorphs, but that is widespread in theropods and other carnivorous archosaurs [6,34]. In addition, the maxillary and dentary tooth crowns of *Pampadromaeus* are slightly recurved, but also expanded at their base, forming a sigmoidal distal margin. This differs from the spear-like tooth crowns of post-Carnian sauropodomorphs [43]. On the other hand, the denticles of *Pampadromaeus* teeth form oblique angles with the margin of the tooth crown, a trait also present in post-Carnian sauropodomorphs, for which a more herbivorous diet is inferred [43].

Despite the presence of few well-preserved teeth, some traits associated with a faunivorous feeding behaviour can be observed in MCP-3845-PV. The denticles are perpendicular to the margin of the tooth crown, as in *Buriolestes* [8,26]. Some of the preserved crowns have a posteriorly curved mesial margin (Figs 10 and 11), although others are convex mesially and distally, with leaf shaped labial/lingual views. Indeed, the presence of such features suggests an at least partially faunivorous feeding behaviour for *Saturnalia* (see also [38]).

The results of the morphological disparity analysis show that the jaw feeding apparatus morphospace of these early Carnian taxa (i.e. *Buriolestes*, *Eoraptor*, *Pampadromaeus*, *Panphagia*, *Saturnalia*) have no overlap with that occupied by later taxa (Fig 13). Thus, even if an omnivorous diet is inferred for some of the Carnian forms such as *Saturnalia*, *Eoraptor*, and *Pampadromaeus*, their feeding behaviour was likely different from that of later omnivorous taxa (see below).

A new late Carnian sauropodomorph, *Bagualosaurus* [11], shows that lanceolate teeth with coarse denticles, as more commonly seen in younger Triassic (e.g. *Efraasia minor*, *Plateosaurus* spp., *Unaysaurus tolentinoi*, *Coloradisaurus brevis*, *Pantydraco caducus*, *Thecodontosaurus antiquus*) and Jurassic (e.g. *Massospondylus* spp. and *Adeopapposaurus mognai*) sauropodomorphs, developed earlier than previously thought. Unlike these later taxa, *Bagualosaurus* has minor or no overlap between tooth crowns, but the results of the morphological disparity analysis show that, even with such differences, the jaw feeding apparatus of *Bagualosaurus* is more similar to that of later sauropodomorphs than to that of other Carnian taxa, expanding the jaw feeding apparatus morphospace of the sauropodomorphs from that time interval (Fig 13).

Except for *Coloradisaurus*, all Norian-Rhaetian sauropodomorphs included in the morphological disparity analysis are non-massopodan plateosaurians. These taxa exhibit a series of characteristics (i.e. coarse denticles, leaf-shaped crowns, overlap between tooth crowns, increase in body mass) that indicate a shift to a more herbivorous diet when compared to that of the bulk of Carnian taxa [43]. The results of the analysis show that the morphospace of *Coloradisaurus* falls into the range of their contemporary taxa rather than into that occupied by its closest relatives (i.e. massopodans). This is not unexpected, given that the skull morphology of *Coloradisaurus* resembles that of non-massopodan plateosaurians, and its placement among massospondylids is mostly supported by postcranial characters [48]. As such, the separation of the ‘Norian-Rhaetian’ and ‘Early Jurassic’ morphospaces observed in our PCoA results cannot be attributed to the phylogenetic position of the taxa within each time interval (Figs 13 and 14). Thus, one possible explanation for this splitting is that changes in the vegetation across the Triassic/Jurassic boundary [49,50] leaded to the shift in morphology seen in the jaw feeding apparatus of Norian-Rhaetian to Early Jurassic taxa (but see [51]–in that study, the results of morphological and biomechanical disparity analyses of the mandible of sauropodomorphs shows an overlap in the morphospace associated with Late Triassic and Early Jurassic taxa). As for the phylogenetic groupings, features of non-anchisaurian massopods such as *Coloradisaurus* and *Yunnanosaurus huangi*, which also has a dentition markedly different from that of its closest relatives [52], contribute to the great morphospace range occupied by that assemblage of sauropodomorphs (Fig 14).

The differentiation between a fully faunivorous and an omnivorous diet cannot rely only on tooth morphology, and the same is true for the differentiation between omnivorous or fully herbivorous taxa [43]. In addition, various Late Triassic and Early Jurassic sauropod or near-sauropod taxa are known only from fragmentary or incomplete cranial materials (e.g. *Vulcanodon karibaensis* [53]; *Lessemsaurus sauropoides* [54]; *Antetonitrus longiceps* [32]; *Pulanesaura eocollum* [55]; *Ingentia prima* [9]). In this context, the acquisition of a fully herbivorous diet in Sauropodomorpha has been associated with the development of a fully quadrupedal stance, with the loss of grasping hands, and the increase in body sizes, enhancing the absorption of nutrients during digestion [42,43]. Prior to recent discoveries, the fossil record of sauropodomorphs indicated that the transition to a fully herbivorous diet was thus more likely to have happened in the Early Jurassic [42,43]. However, recent findings have shown that sauropodomorph gigantism predates the Triassic-Jurassic boundary [9,10], with the transition to fully herbivorous diets, at least in some lineages, having occurred still during the Triassic. In this context, the relatively small jaw feeding apparatus morphospace range associated with Late Triassic forms in comparison to that of Early Jurassic taxa might be an artefact of the paucity of cranial material associated with those large Late Triassic sauropodomorphs (see also [56]). Nevertheless, the occupation of a large jaw feeding apparatus morphospace by Early Jurassic sauropodomorphs can be interpreted as an additional evidence of niche partitioning, as previously suggested based on the differences in their postcranial skeleton [55]. Thus, a stronger correlation between dental and postcranial adaptations towards herbivory may have also been recorded for the Late Triassic if a larger and more even specimen sample was known.

Finally, it is also important to stress some of the caveats associated with the results of our PCoA analyses (see also [57]). The morphospace occupied by a set of taxa grouped based both on temporal distribution (Fig 13) and phylogenetic position (Fig 14), varies largely across graphs based on different PCos (S3 Appendix). For instance, the separation between Late Triassic and Early Jurassic taxa is recovered (i.e. visually assessed) when PCo1 is plotted against PCo2 (Fig 13), but not when some other PCos are plotted against one another (S3 Appendix). Additionally, the morphospace of the sauropodomorphs more closely related to *Saturnalia tupiniquim* than to *Bagualosaurus agudoensis* shows some overlap with those of other clades when PCo1 is plotted against PCo3 (but not when PCo2 is plotted against PCo3). Nevertheless, the results of the npMANOVA, which was conducted taking information on all PCO’s into account, indicate that the separations observed between the groups (both the temporal and phylogenetic groups here defined) when PCo1 is plotted against PCo2 are statistically significant.

## Conclusions

**Fig 15:**
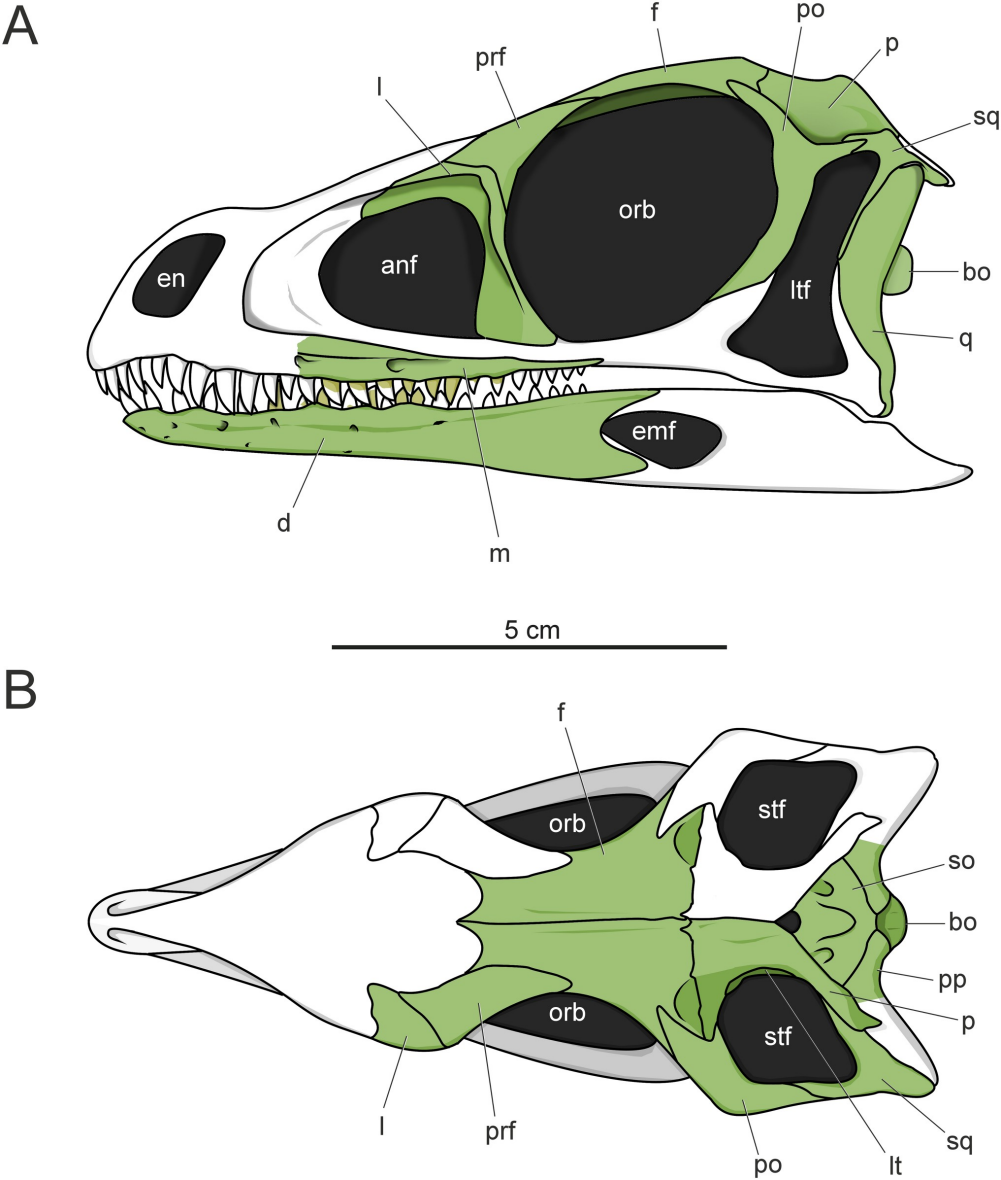
Reconstruction of the skull of *Saturnalia tupiniquim* in lateral (A) and dorsal (B) views, with preserved bones highlighted in green. Abbreviations: **anf—**antorbital fenestra; **bo—**basioccipital; **d—**dentary; **emf—**external mandibulary fenestra; **en—**external naris; **f—**frontal; **l—**lacrimal; **lt—**laterosphenoid; **ltf—**laterotemporal fenestra; **m—**maxilla; **orb—**orbit; **p—**parietal; **po—**postorbital; **pp—**paroccipital process; **prf—**prefrontal; **q**—quadrate; **so—**supraoccipital; **sq—**squamosal; **stf—**supratemporal fenestra.

Part of the osteology of the Carnian (Late Triassic) dinosaur *Saturnalia* has been described in a series of papers [3,7,15,38,58]. Here we provide the first detailed description of the only skull cover elements known for the taxon (Fig 15), which belong to one of the paratypes (MCP-3845-PV), filling a gap on our knowledge on the anatomy of this taxon. The newly described material provides stronger support for the inference that *Saturnalia* possesses a reduced skull, typical of later sauropodomorphs. Furthermore, when this information is analysed alongside other aspects, including dental and brain-tissues anatomy, it suggests that the initial skull reduction of sauropodomorphs might have been indeed associated with an increase in efficiency of a predatory lifestyle as previously suggested [33].

The description presented here will be source for future phylogenetic and/or comparative studies dealing with the early evolution of dinosaurs and sauropodomorphs. The results of the two phylogenetic analyses highlight the uncertainty on the relations among Carnian sauropodomorphs, with the hypotheses showing differences on the affinity of taxa such as *Buriolestes*, *Chromogisaurus*, *Eoraptor*, *Pampadromaeus*, *Panphagia*, and *Saturnalia* (see also [26]). Thus, the hypothesis that features associated with the acquisition of a more omnivorous/herbivorous diet appeared in a stepwise fashion among sauropodomorphs does not fit to all possible phylogenetic scenarios. Nevertheless, the dental anatomy of *Saturnalia* adds evidence that the group achieved a relatively broad ecomorphological disparity in the Carnian, what might explain their higher diversity when compared to other dinosaur groups in the oldest dinosaur-bearing strata in South America.

Based on the new data from *Saturnalia* and other recently discovered taxa, the early evolution of the sauropodomorph diet can be traced in more detail. Carnian sauropodomorphs show great variety in tooth morphology, but still seemingly correspond to at least partially faunivorous taxa. One exception is the recently discovered *Bagualosaurus* [11], the tooth and postcranial anatomy (i.e. increase in body size) of which shows that a shift to a more herbivorous lifestyle have happened still during the Carnian. Our PCoA analysis reveals marked shifts in the morphology of the jaw features associated with the feeding behaviour through time. These shifts are ultimately linked to transformations in the postcranial anatomy (see also [9,10]), and highlight that the first 55 million years of sauropodomorph evolution, from the Late Triassic (Carnian–c. 230 Ma) to the last stages of the Early Jurassic (Toarcian–c. 175 Ma), were marked by important changes in the ecomorphology of the group.

## Supporting information

S1 Appendix Details of the phylogenetic analyses presented in this study. (DOCX)

S2 Appendix Part of the skull roof of *Saturnalia tupiniquim* MCP-3845-PV in dorsal view. (JPG)

S3 Appendix Data matrix and additional results of the principal co-ordinates analysis. (PDF)

## Abbreviations

AMNH: American Museum of Natural History, New York, USA
CAPPA/UFSM: Centro de Apoio à Pesquisa Paleontológica, Universidade Federal de Santa Maria, Santa Maria, Brazil
MCP PV: Museu de Ciências e Tecnologia, Pontificia Universidade Católica do Rio Grande do Sul, Porto Alegre, Brazil
PULR: Universidad Nacional de La Rioja, La Rioja, Argentina
PVSJ: Museo de Ciencias Naturales, San Juan, Argentina
SAM: Iziko South African Museum, Capetown, South Africa
SMNS: Staatliches Museum für Naturkunde, Stuttgart, Germany
