# Supplementary figures and images for: Skull remains of the dinosaur Saturnalia tupiniquim (Late Triassic, Brazil): With comments on the early evolution of sauropodomorph feeding behaviour
Source: PLoS One. 2019 Sep 6;14(9):e0221387. doi: 10.1371/journal.pone.0221387 (PMC6730896; doi:10.1371/journal.pone.0221387)

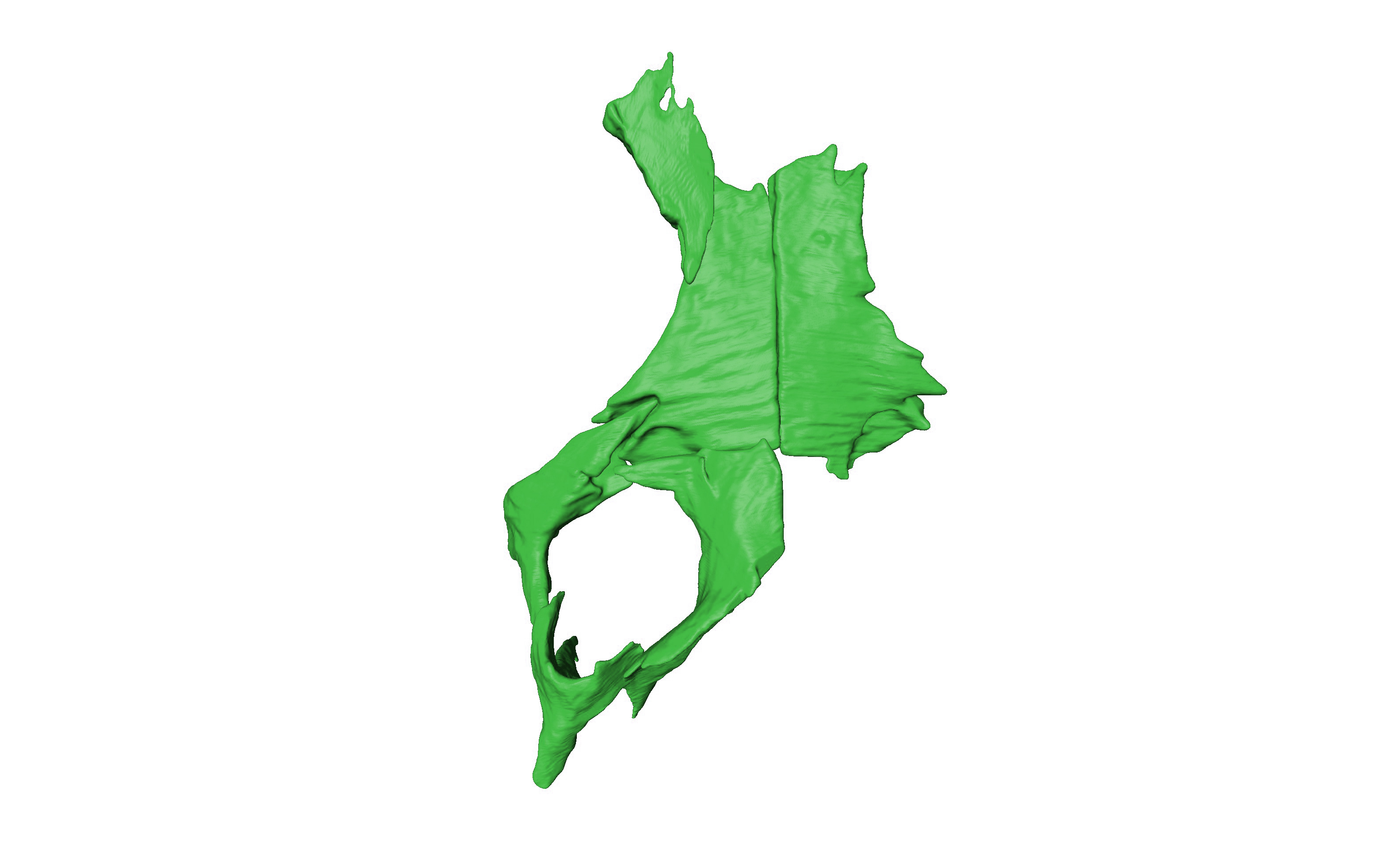

Supplement: S2 Appendix — (JPG) [file pone.0221387.s002.jpg]
