# Supplementary material for: Skull remains of the dinosaur Saturnalia tupiniquim (Late Triassic, Brazil): With comments on the early evolution of sauropodomorph feeding behaviour
Source: PLoS One. 2019 Sep 6;14(9):e0221387. doi: 10.1371/journal.pone.0221387 (PMC6730896; doi:10.1371/journal.pone.0221387)
Supplement: S3 Appendix — (PDF) [file pone.0221387.s003.pdf]

```
#NEXUS

[written Tue Nov 20 17:04:57 BRST 2018 by Mesquite  version 3.31 (build 859) at MarioPC/10.59.133.238]


BEGIN TAXA;

	TITLE Taxa;

	DIMENSIONS NTAX=28;

	TAXLABELS

		Bagualosaurus Eoraptor Panphagia Saturnalia Buriolestes Pampadromaeus Aardonyx Adeopapposaurus Anchisaurus Coloradisaurus Efraasia Jingshanosaurus Leyesaurus Lufengosaurus Mamenchisaurus Massospondylus2 NMQR3314 Neosauropoda Omeisaurus Pantydraco Patagosaurus Plateosaurus_engelhardti Plateosaurus_gracilis Riojasaurus Shunosaurus Tazoudasaurus Thecodontosaurus Unaysaurus Yunnanosaurus

	;


END;




BEGIN CHARACTERS;

	TITLE  Character_Matrix;

	DIMENSIONS  NCHAR=37;

	FORMAT DATATYPE = STANDARD GAP = - MISSING = ? SYMBOLS = "  0 1 2 3";

	MATRIX

	Bagualosaurus           01001010???01?0010000?001011111110001

	Eoraptor               0000001000?0?00?1000000001100010000?0

	Panphagia              ?1?00010000?11?010000000??11?1011??0?

	Saturnalia             0??00000????000010000000??1??100???1?

	Buriolestes            0100001000001000100000000111100000100

	Pampadromaeus          0??0000000001100100000000(01)?111111110

	Aardonyx               0?00?1?0???11?001001100110000?11?????

	Adeopapposaurus        0001011(01)10101111110(01)10011010011111001

	Anchisaurus            01010?00100000000010?1(12)?011?1????11??01

	Coloradisaurus         01010110011??00010010001?01?11111??0?
```

```
	Efraasia              0101001011?0110010010000????11?1????1
	Jingshanosaurus        01010010111011001021100 1?0???1?1????1
	Leyesaurus            000101101010?111100(01)0001100???11111??
	Lufengosaurus          11010???111?110110010001???0(01)111?????
	Mamenchisaurus         011000011000111111112111000001?1???01
	Massospondylus2        010101101010110(01)10011001100001111?0?(01)
	NMQR3314               110?00111000110?1021100110?00110111?(01)
	Neosauropoda           011000011000(01)11111(02)12(01)11000001?1???01
	Omeisaurus             01100001?00011101111211100??01111??01
	Pantydraco             000010100000?10010010000??1??111?1011
	Patagosaurus           ??100001????0??011012111???????1?????
	Plateosaurus_engelhardti  0101011011111100100100001010111110 0(01)1
	Plateosaurus_gracilis    0?0?0110???1110010010?0010???1111????
	Shunosaurus            00100001100011111212111000?10?1???01
	Tazoudasaurus          ?0000000?0??10?111012101??0??0111????
	Thecodontosaurus       00011010????110010010000?????0110??1?
	Unaysaurus             01010110???0110010010000???0?0111????
	Yunnanosaurus          01000010?0???0001021000??0??00???11??

;

END;
BEGIN ASSUMPTIONS;
	TYPESET * UNTITLED  =  ord:  1 -  37;

	EXSET * UNTITLED  = ;

	WTSET * UNTITLED  =  1: 1 -  37 ;

END;
```

```
BEGIN MESQUITECHARMODELS;
	ProbModelSet * UNTITLED  =  'Mk1 (est.)':  1 -  37;
END;


Begin MESQUITE;
		MESQUITESCRIPTVERSION 2;
		TITLE AUTO;
		tell ProjectCoordinator;
		timeSaved 1542740697446;
		getEmployee #mesquite.minimal.ManageTaxa.ManageTaxa;
		tell It;
			setID 0 399019071671106162;
		endTell;
		getEmployee #mesquite.charMatrices.ManageCharacters.ManageCharacters;
		tell It;
			setID 0 2730348528401152121;
			mqVersion 331;
			checksumv 0 3 975685228 null  getNumChars 37 numChars 37 
getNumTaxa 28 numTaxa 28   short true   bits 7   states 7   sumSquaresStatesOnly 2242.0 
sumSquares 2242.0 longCompressibleToShort false usingShortMatrix true   NumFiles 1 
NumMatrices 1;
			mqVersion;
		endTell;
		getWindow;
		tell It;
			suppress;
			setResourcesState false false 100;
			setPopoutState 300;
			setExplanationSize 0;
			setAnnotationSize 0;
			setFontIncAnnot 0;
			setFontIncExp 0;
```

```
setSize 1354 649;
setLocation -8 0;
setFont SanSerif;
setFontSize 10;
getToolPalette;
tell It;
endTell;
desuppress;
endTell;
getEmployee #mesquite.charMatrices.BasicDataWindowCoord.BasicDataWindowCoord;
tell It;
showDataWindow #2730348528401152121 #mesquite.charMatrices.BasicDataWindowMaker.BasicDataWindowMaker;
tell It;
getWindow;
tell It;
setExplanationSize 30;
setAnnotationSize 20;
setFontIncAnnot 0;
setFontIncExp 0;
setSize 1254 577;
setLocation -8 0;
setFont SanSerif;
setFontSize 10;
getToolPalette;
tell It;
setTool mesquite.charMatrices.BasicDataWindowMaker.BasicDataWindow.arrow;
endTell;
setActive;
```

```
setTool
mesquite.charMatrices.BasicDataWindowMaker.BasicDataWindow.arrow;
colorCells
#mesquite.charMatrices.ColorByState.ColorByState;
tell It;
setStateLimit 9;
toggleUniformMaximum on;
endTell;
colorRowNames
#mesquite.charMatrices.TaxonGroupColor.TaxonGroupColor;
colorColumnNames
#mesquite.charMatrices.CharGroupColor.CharGroupColor;
colorText  #mesquite.charMatrices.NoColor.NoColor;
setBackground White;
toggleShowNames on;
toggleShowTaxonNames on;
toggleTight off;
toggleThinRows off;
toggleShowChanges on;
toggleSeparateLines off;
toggleShowStates on;
toggleAutoWCharNames on;
toggleAutoTaxonNames off;
toggleShowDefaultCharNames off;
toggleConstrainCW on;
toggleBirdsEye off;
toggleShowPaleGrid off;
toggleShowPaleCellColors off;
toggleShowPaleExcluded off;
togglePaleInapplicable on;
toggleShowBoldCellText off;
toggleAllowAutosize on;
```

```
				toggleColorsPanel off;
				toggleDiagonal on;
				setDiagonalHeight 80;
				toggleLinkedScrolling on;
				toggleScrollLinkedTables off;
			endTell;
			showWindow;
			getWindow;
			tell It;
				forceAutosize;
			endTell;
			getEmployee #mesquite.charMatrices.AlterData.AlterData;
			tell It;
				toggleBySubmenus off;
			endTell;
			getEmployee #mesquite.charMatrices.ColorCells.ColorCells;
			tell It;
				setColor Red;
				removeColor off;
			endTell;
			getEmployee
#mesquite.categ.StateNamesStrip.StateNamesStrip;
			tell It;
				showStrip off;
			endTell;
			getEmployee #mesquite.charMatrices.AnnotPanel.AnnotPanel;
			tell It;
				togglePanel off;
			endTell;
			getEmployee
#mesquite.charMatrices.CharReferenceStrip.CharReferenceStrip;
			tell It;
```

```
					showStrip off;
				endTell;
				getEmployee
#mesquite.charMatrices.QuickKeySelector.QuickKeySelector;
				tell It;
					autotabOff;
				endTell;
				getEmployee
#mesquite.charMatrices.SelSummaryStrip.SelSummaryStrip;
				tell It;
					showStrip off;
				endTell;
				getEmployee
#mesquite.categ.SmallStateNamesEditor.SmallStateNamesEditor;
				tell It;
					panelOpen true;
				endTell;
			endTell;
		endTell;
		endTell;
end;
```

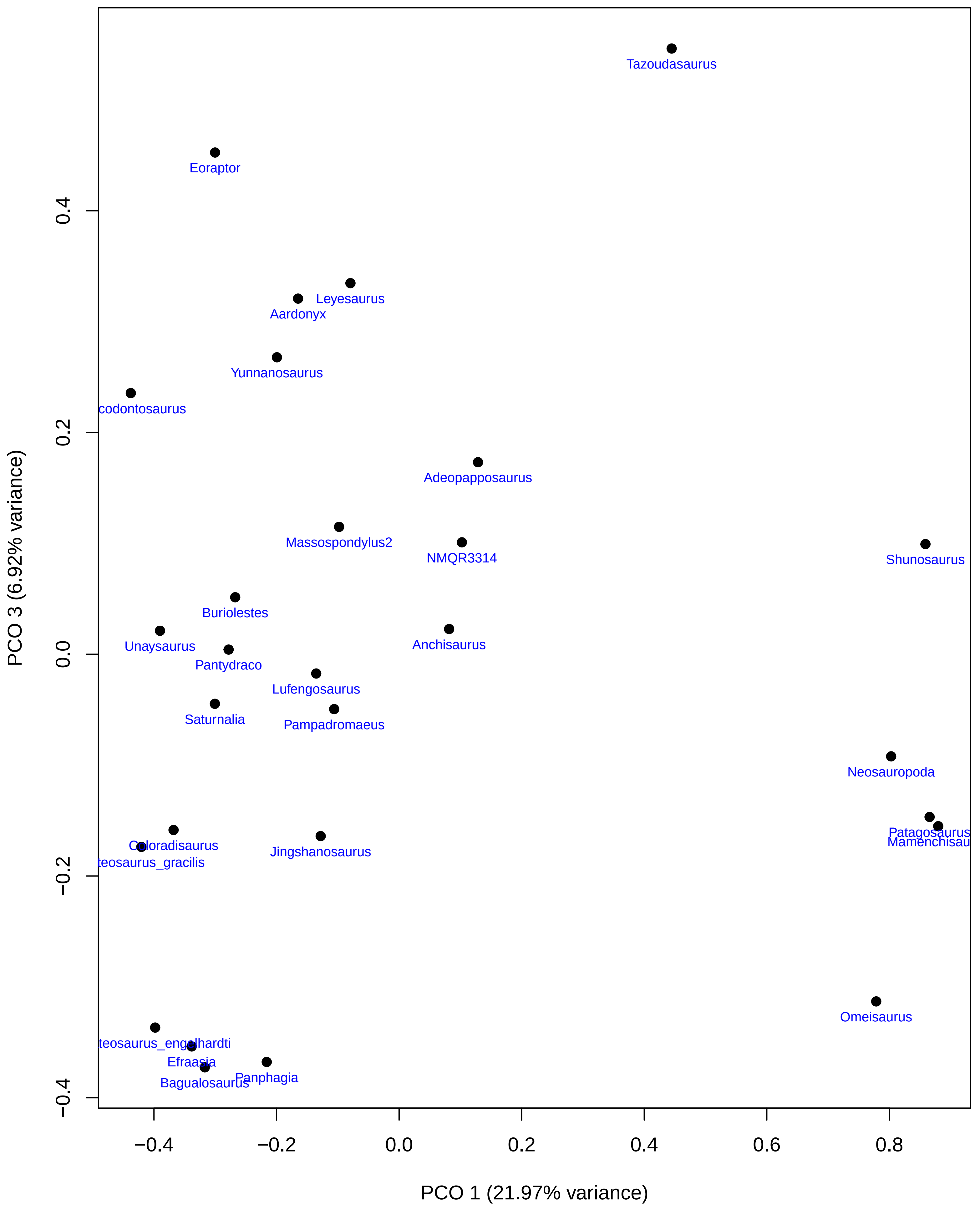

Tazoudasaurus
Eoraptor
Leyesaurus
Aardonyx
Yunnanosaurus
codontosaurus
Adeopapposaurus
Massospondylus2
NMQR3314
Shunosaurus
Buriolestes
Unaysaurus
Pantydraco
Anchisaurus
Lufengosaurus
Saturnalia
Pampadromaeus
Neosauropoda
Patagosaurus
Mamenchisau
Coloradisaurus
teosaurus_gracilis
Jingshanosaurus
Omeisaurus
teosaurus_engelhardti
Efraasia
Panphagia
Bagualosaurus
PCO 3 (6.92% variance)
PCO 1 (21.97% variance)
−0.4
−0.2
0.0
0.2
0.4
0.6
0.8

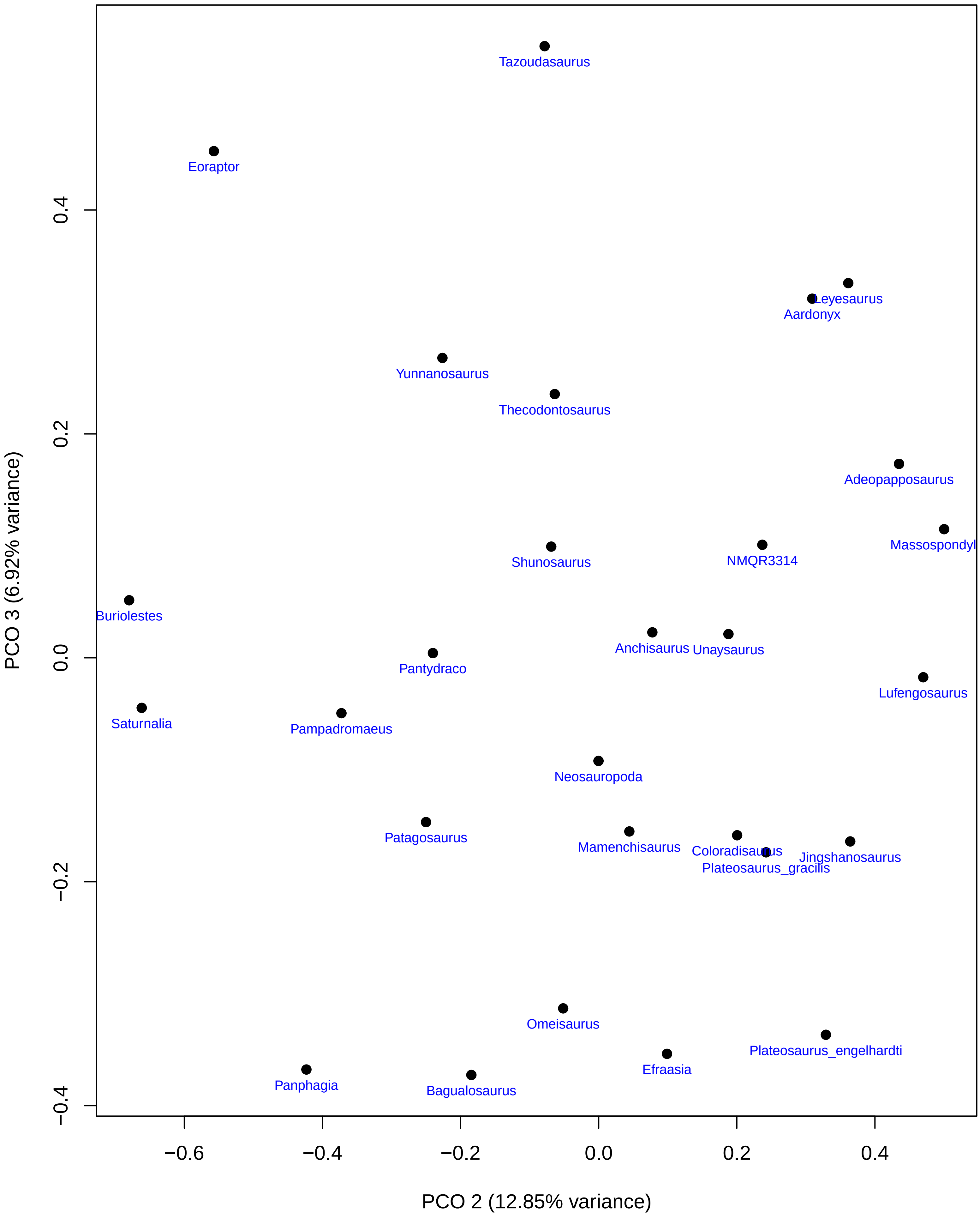
Tazoudasaurus
Eoraptor
Leyesaurus
Aardonyx
Yunnanosaurus
Thecodontosaurus
Adeopapposaurus
Massospondyl
Shunosaurus
NMQR3314
Buriolestes
Anchisaurus
Unaysaurus
Pantydraco
Lufengosaurus
Saturnalia
Pampadromaeus
Neosauropoda
Patagosaurus
Mamenchisaurus
Coloradisaurus
Jingshanosaurus
Plateosaurus_gracilis
Omeisaurus
Plateosaurus_engelhardti
Efraasia
Panphagia
Bagualosaurus
PCO 3 (6.92% variance)
PCO 2 (12.85% variance)
0.4
0.2
0.0
−0.2
−0.4
−0.6
−0.4
−0.2
0.0
0.2
0.4
